# Tri-modal nanocatalytic microenvironment regulations for macrophage reprogramming and osteoporotic fracture healing promotion

**DOI:** 10.1016/j.mtbio.2026.103389

**Published:** 2026-06-23

**Authors:** Bo Yuan, Jia Fu, Yin Zhao, Gang Zheng, Han Lin, Xiongsheng Chen, Xiang Guo, Jianlin Shi

**Affiliations:** a Department of Orthopedics, Shanghai General Hospital, School of Medicine, Shanghai Jiao Tong University, Shanghai, 200080, China; b Department of Orthopaedics, Shanghai Changzheng Hospital, Second Affiliated Hospital of Naval Medical University, Shanghai, 200003, China; c State Laboratory of High Performance Ceramics and Superfine Microstructure, Shanghai Institute of Ceramics, Chinese Academy of Sciences, Shanghai, 200050, China; d School of Health Science and Engineering, University of Shanghai for Science and Technology, Shanghai, 200093, China

**Keywords:** Osteoporotic fracture, Nanocatalytic medicine, Macrophage polarization, Metabolic reprogramming, Mitochondrial remodeling

## Abstract

The clinical challenge of osteoporotic fracture healing is rooted in a hostile local microenvironment characterized by oxidative stress, hypoxia, and acidosis, which stalls regeneration by trapping macrophages in a pro-inflammatory, metabolically crippled state. Herein, we report an intelligent nanocatalytic medicine composed of a pH-responsive calcium-aluminum layered double hydroxide (CaAl-LDH) decorated with a reactive oxygen species (ROS)-responsive manganese oxide (MnOx), denoted as CALM, for orthopedic implantation. This hierarchical system is designed to modulate key features of the “triple threat” microenvironment: the CaAl-LDH backbone dissolves in the local microenvironment to buffer the acidity, while the MnOx scavenges ROS and simultaneously generates therapeutic oxygen. This comprehensive microenvironment modulation supports mitochondrial function and drives the metabolic and phenotypic reprogramming of macrophages from a pro-inflammatory M1 to a pro-reparative M2 state. In a clinically relevant osteoporotic rat fracture model, the CALM coating significantly accelerated bone regeneration. Mechanistically, transcriptomic and protein-level analyses reveal that CALM exerts its immunomodulatory and osteogenic effects by activating the PI3K/Akt/GSK3β signaling pathway. This work presents a metabolically focused, nano-enabled strategy to break the cycle of non-union, offering a promising therapeutic platform for the treatment of osteoporotic fractures.

## Highlights

• A pH/ROS-responsive CALM coating was developed for osteoporotic fracture repair.
• CALM buffers acidity and modulates ROS- and hypoxia-related cellular stress.
• CALM promotes M2-like macrophage reprogramming via metabolic regulation.
• CALM-conditioned macrophage media enhance BMSC osteogenic differentiation.
• CALM coating significantly accelerates bone regeneration in osteoporotic rats.

## Graphical abstract

**Figure:**
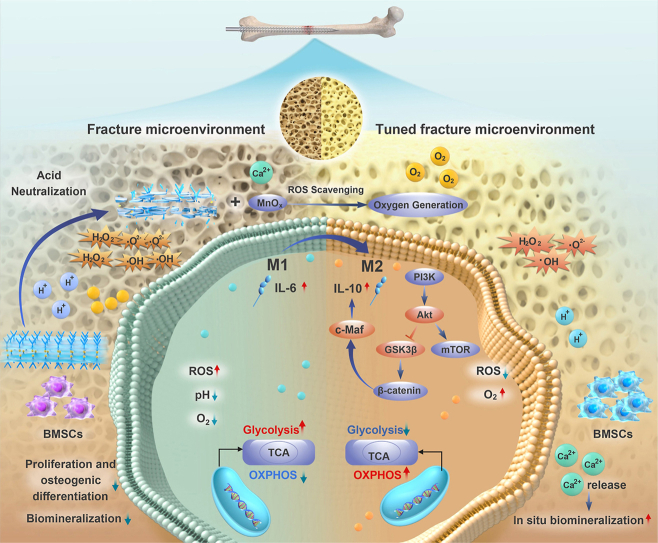

## 1 Introduction

Osteoporosis is a systemic skeletal disease characterized by diminished bone mineral density and microarchitectural deterioration, which has emerged as a formidable global health crisis [1]. The concomitant increase in bone fragility leads to a high incidence of osteoporotic or fragility fractures, which represent a significant source of morbidity, disability, and mortality, particularly among the aging population [2,3]. The clinical management of these fractures presents a profound orthopedic challenge. Although the surgical internal fixation techniques are adept at providing the mechanical stability essential for bone healing, they frequently encounter a relatively high failure rate in the context of osteoporosis due to a profoundly compromised biological healing capacity, rooted in an imbalance between bone formation and resorption [4]. More fundamentally, a harsh microenvironment of high oxidative stress state at fracture site exacerbates the damage, resulting in a prolonged and often incomplete regenerative process with an alarmingly high rate of delayed union and non-union, ranging from 15% to 41% [5,6]. To address this biological gap, intervention to correct the underlying biological deficits that impede healing in the osteoporotic host shows its necessity.

At the site of a traumatic injury, a “triple threat” of pathological conditions converges: excessive oxidative stress, pervasive hypoxia, and an inhibitory acidic milieu [7]. This extreme oxidative stress, driven by ischemia-reperfusion injury and the metabolic activity of infiltrating inflammatory cells, is a core pathological driver of the bone metabolic imbalance seen in osteoporosis [[8], [9], [10]]. The overabundance of ROS like hydrogen peroxide (H_2_​O_2_​) and superoxide anions (O_2_•^-^) inflict direct damage on the lipids, proteins, and DNA of critical bone-forming cells, thereby impairing their function, inducing apoptosis, and fundamentally hindering the synthesis of new bone matrix [11,12]. Concurrently, vascular disruption creates a severe hypoxic environment that forces repair-related cells into inefficient anaerobic glycolysis, stalling the energy-intensive healing process [[13], [14], [15], [16]]. This metabolic shift, coupled with tissue necrosis, generates an acidic milieu that further inhibits mineralization and cell viability [17].

Central to this pathological cascade is the dysregulation of macrophages, the key orchestrators of bone repair [8,18]. These highly plastic immune cells are the master regulators of tissue repair, capable of adopting distinct functional phenotypes in response to environmental cues. In healthy healing, a tightly regulated transition from a transient pro-inflammatory (M1) phase to a pro-reparative (M2) phenotype is essential [19]. In the context of osteoporotic fractures, this crucial M1-to-M2 transition fails [20,21]. The persistent oxidative, hypoxic, and acidic microenvironment is fundamentally incompatible with the M2 phenotype, locking macrophages in a chronic pro-inflammatory M1 state, where they promote bone resorption and suppress osteoblast function [22,23]. Recent advances in immunometabolism reveal that this phenotypic arrest is intrinsically governed by profound shifts in cellular metabolism [24]. The M1 and M2 phenotypes are underpinned by distinct metabolic signatures [25,26]. Pro-inflammatory M1 activation is fueled by aerobic glycolysis, a metabolic program that enables the rapid production of inflammatory mediators but is inefficient for sustained cellular processes. In stark contrast, the transition to the pro-reparative M2 phenotype is metabolically demanding and requires a fundamental switch to mitochondrial-dependent pathways [27,28]. This reframes the clinical problem of non-union as a failure of metabolic reprogramming. Therefore, creating a permissive microenvironment to rescue the metabolic capacity of the host's own immune cells represents a primary therapeutic target.

**Scheme 1:**
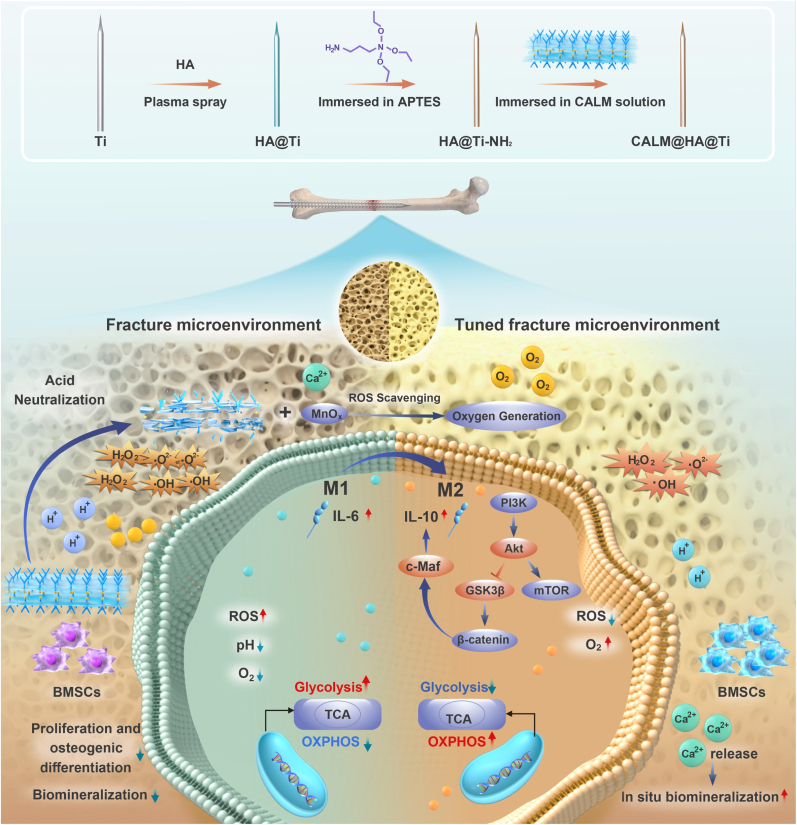
Schematic illustration of the synthesis process of the CALM@HA composite coating and the mechanism of metabolic reprogramming of macrophages by a microenvironment-modulating.

Recent advancements in catalytic nanomedicine have highlighted the tremendous potential of microenvironment-responsive nanozymes and single-atom catalysts in reshaping pathological immune niches. For instance, strategically engineered manganese- or ruthenium-based nanozymes have demonstrated remarkable efficacy in reprogramming macrophages and regulating redox homeostasis for infected wound healing and intracerebral hemorrhage therapy [[29], [30], [31], [32], [33], [34]]. Layered double hydroxide (LDH) is a type of two-dimensional nanomaterial composed of octahedral host layers and interlayer negatively charged anions [35]. Due to its high adjustability (size, metal cations and interlayer anions), interlayer loadability and good biocompatibility, LDH has received extensive attention in biomedical fields such as tumor diagnosis and treatment, bioimaging and drug delivery [[36], [37], [38]]. Herein, we constructed a multi-functional nanocatalytic implant coating by synthesizing calcium-aluminum-LDH functionalized with manganese oxide (Ca-Al-LDH-MnOx, termed CALM). This coating was engineered to respond to local pH and ROS levels and to modulate key features of the hostile fracture microenvironment, thereby promoting macrophage reprogramming and osteoporotic fracture healing. Crucially, CALM represents a rationally designed system featuring complementary physicochemical and biological synergies. Physicochemically, the CaAl-LDH backbone degrades in response to low pH, neutralizing the local acidity. This localized acid-buffering effectively prevents the premature burst dissolution of MnOx, creating a permissive microenvironment that sustains its catalytic durability. Biologically, the MnOx nanocatalysts scavenge endogenous ROS and generate therapeutic oxygen (O_2_​), helping preserve macrophage mitochondrial function and supporting a metabolic shift from glycolysis to oxidative phosphorylation. Concurrently, the acid-triggered release of Ca^2+^ from the LDH framework *in situ* forms calcium phosphate nanoparticles, which independently promote M2 macrophage polarization through c-Maf activation [36]. The MnOx-driven immunometabolic reprogramming and Ca^2+^-driven c-Maf activation jointly promote macrophage polarization toward a pro-reparative M2-like phenotype (Scheme 1). As expected, the in vitro and in vivo results suggest that the CALM composite coating promotes macrophage reprogramming and osteoporotic fracture healing through ROS scavenging, oxygen-generating activity, and acid-neutralizing capacity. Moreover, transcriptome sequencing analysis conducted on RAW264.7 macrophages revealed that CALM-mediated osteogenesis enhancement may occur partly through activation of PI3K/Akt/GSK3*β* pathway in macrophages, which may in turn create a pro-osteogenic microenvironment for osteogenic precursor cells. This work introduces a single, hierarchical system for smart implant coatings, offering a promising therapeutic strategy to address the profound clinical challenge of osteoporotic fracture.

## 2 Results and discussion

### 2.1 Synthesis and characterization of 2D CALM nanosheets

**Fig. 1:**
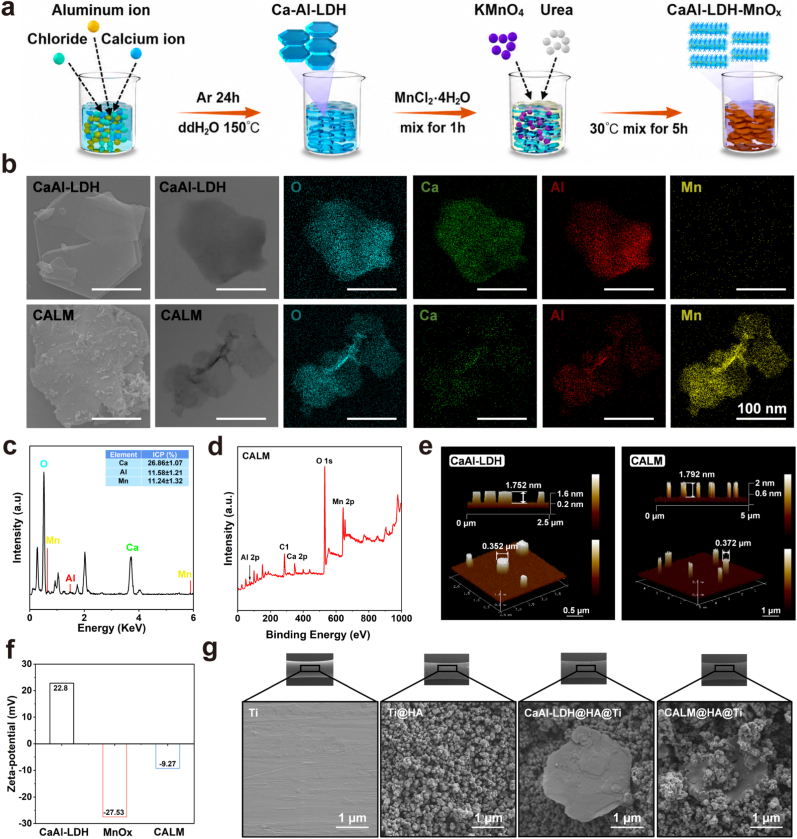
Synthesis and characterization of 2D CALM nanosheets. a) Schematic illustration of the synthesis process of the CALM. b) SEM and EDS elemental mapping of CaAl-LDH and CALM (O, Ca, Al, Mn). c) Elemental analyses of CALM by EDS and ICP-OES (inset table). d) XPS spectra of CALM. e) AFM image of CALM. f) Zeta potentials of CALM. g) Digital photos and SEM images of Ti, HA@Ti, CaAl-LDH@HA@Ti, and CALM@HA@Ti, respectively. Scale bar: 100 nm (EDS images) and 1 μm (AFM and SEM images).

The synthesis and characterization of the 2D CALM nanosheets are presented in Fig. 1. Initially, CaAl-LDH nanosheets were fabricated via a homogeneous alkalization method, consistent with previous reports [36,39,40]. To impart stimuli-responsive properties for post-traumatic applications, these nanosheets were subsequently decorated with MnOx via in-situ synthesis method (Fig. 1a). The morphology and elemental composition of CALM were thoroughly investigated. The scanning electron microscopy (SEM) image of CALM in Fig. 1b shows that the LDH-based nanosheets have a hexagonal shape. In Fig. 1c, the successful incorporation of manganese was confirmed by energy-dispersive X-ray spectroscopy (EDS), with corresponding elemental mappings showing existence of Ca, Al, and Mn throughout the nanosheet structure (Fig. 1b). Quantitative analysis by inductively coupled plasma optical emission spectrometry (ICP-OES) determines the mass percentage of Ca, Al, and Mn elements in the CALM nanosheets to be 26.86%, 11.58%, and 11.24%, respectively (Fig. 1c). X-ray photoelectron spectroscopy (XPS) was employed to investigate the chemical state of CALM, and the resultant Ca 2p, Al 2p and Mn 2p spectra are shown in Fig. 1d. The fitting peaks located at 347.24 and 350.81 eV correspond to Ca 2p3/2 and Ca 2p1/2 orbitals, showing the presence of Ca^2+^ species in CALM (Fig. S1a) [41,42]. The fitting peak located at 74.2 eV is ascribed to Al 2p (Fig. S1b), indicating the presence of Al^3+^ species in CALM. Critically, the Mn 2p peaks are at 640.87 and 652.57 eV, corresponding to the Mn 2p_3_/_2_ and Mn 2p_1_/_2_ orbitals, respectively (Fig. S1c) [43]. These binding energies are indicative of the Mn^4+^ oxidation state, validating the successful formation of MnOx. Furthermore, Fig. S1e shows that the O1s band can be deconvoluted into two sub-bands: “metal-O” (Al-O, 530.47 eV) and “O2-oxo-species” (528.46 eV) [44]. The X-ray diffraction (XRD) patterns of CaAl-LDH and CALM both display characteristic (003) diffraction peaks indicative of a layered double hydroxide structure (Fig. S2).

The physical dimensions of the nanosheets were also characterized. Atomic force microscopy (AFM) was used to confirm the 2D nature of CALM nanosheets, revealing a thickness of approximately 1.5–3 nm (Fig. 1e, Fig. S3b). The as-prepared CALM nanosheets exhibit a lateral size of approximately 300 nm (Fig. S3a), which feature the typical 2D standalone morphology. In Fig. S4, the peak particle size is 164.7 nm and has an excellent dispersity in ethanol solution. Furthermore, the transmission electron microscopy (TEM) image and digital photos showed that CALM was 200 nm in lateral dimension and had an excellent dispersion (Fig. S5).

To facilitate stable immobilization of CALM nanosheets onto an implant, an electrostatic adsorption strategy was developed. The zeta-potential measurements showed that MnOx modification shifted the surface charge from approximately +20 mV for CaAl-LDH to −10 mV for CALM (Fig. 1f), facilitating electrostatic interactions with positively charged -NH2 groups on the HA@Ti surface. This significant charge reversal is primarily attributed to the dense deposition of negatively charged MnOx nanoparticles, which effectively neutralize and mask the intrinsic positive charges of the LDH lamellar host layers. SEM observations confirmed the successful coating of CALM nanosheets onto HA-coated Kirschner wires (Fig. 1g). The implant's surface was first prepared by plasma-spraying a hydroxyapatite (HA) layer, followed by a brief treatment with diluted HCl, which rendered the surface relatively rough to enhance adhesion. A roughened surface was then functionalized with 3-aminopropyltriethoxysilane (APTES) to introduce positively charged ammonium groups. As expected, Fig. 1g suggests that immersion of this positively-charged substrate into the negatively-charged CALM dispersion resulted in the uniform anchoring of the platelet-like CALM nanosheets onto the HA coating through strong electrostatic interactions.

### 2.2 In vitro ROS scavenging, oxygen generation, and acid neutralization

**Fig. 2:**
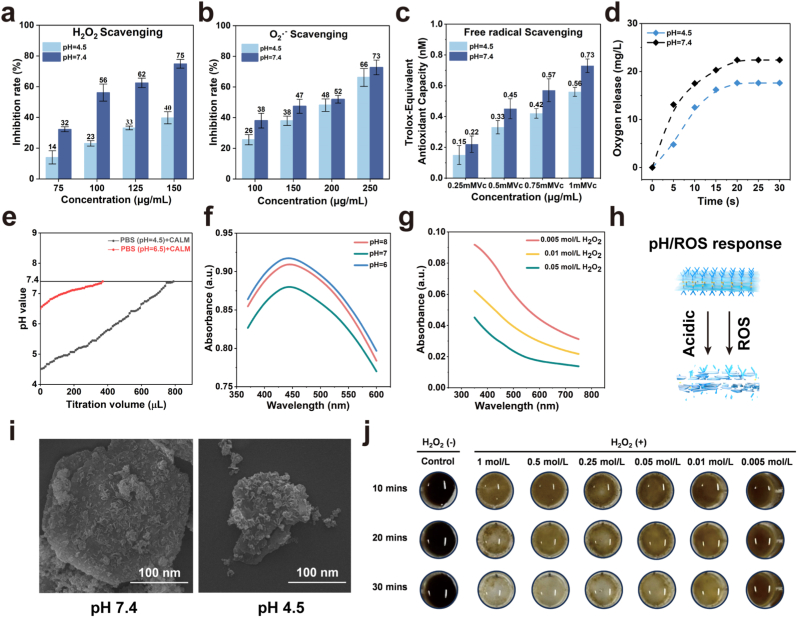
In vitro ROS scavenging, oxygen generation, and acid neutralization capacity evaluation. a-c) ROS-scavenging activities of CALM nanosheets toward multiple free radicals including H_2_O_2_, O_2_•^-^, ABTS^+•^. d) Oxygen-generation curves of CALM at different pH values. e) pH monitoring during the titration of CALM into acidic PBS (pH 4.5 or 6.5). f, g) Absorbance of CALM in PBS of varied pH values or H_2_O_2_ concentrations. h) Scheme of microenvironment-responsive biodegradation of CALM. i) SEM of CALM in PBS of varied pH values. j) Photographs of CALM nanosheets dispersed in PBS solutions containing different H_2_O_2_ concentrations. Data in (a), (b) and (c) were presented as mean values ± S.D. (n = 4).

The post-traumatic microenvironment is characterized by a detrimental triad of high oxidative stress, local hypoxia, and acidosis, which collectively impair bone regeneration, particularly in osteoporotic conditions [7]. In such patients, excessive ROS accumulation activates the pro-inflammatory NF-κB pathway, suppresses the Nrf2 antioxidant system, and promotes M1 macrophage polarization [45] and mitochondrial dysfunction [46]. Therefore, an implant with potent capabilities to neutralize these pathological cues is highly desirable. The ROS-scavenging efficacy of CALM nanosheets was quantitatively investigated. First, we measured the consumption rate of CALM nanosheet in the presence of H_2_O_2_ under different pH conditions (Fig. 2a). The inhibition rate demonstrates a positive correlation with the CALM concentration in both acidic and neutral environments. Notably, the H_2_O_2_ inhibition rate of 75% was achieved at a CALM concentration of 150 μg mL^−1^. We further assessed its performance against O_2_•^-^, a key mediator of oxidative damage. Treatment with 250 μg mL^−1^ of CALM resulted in a 73% depletion of O_2_•^-^ (Fig. 2b). To evaluate its total antioxidant capacity, a classic ABTS radical cation decolorization assay was performed. The results indicated that CALM nanosheets effectively reduced ABTS^+•^ radicals, contributing to marked removal (0.73 nM) of free radicals at a concentration of 1 mM Vc (Fig. 2c, Fig. S6), highlighting their broad-spectrum antioxidant activity. These findings establish CALM as a potent scavenging agent for detrimental ROS, capable of fostering a pro-regenerative microenvironment for fracture repair.

The hypoxic and ROS-rich post-fracture microenvironment forces macrophages and bone marrow stromal cells (BMSCs) into anaerobic glycolysis, which inhibits M2 macrophage polarization and osteogenic differentiation, thereby delaying healing [47]. Restoring oxygen levels is crucial, as the anti-inflammatory and osteogenic functions of these cells rely on aerobic metabolism to meet high energy demands [15,16]. We therefore examined the oxygen-generating capability of CALM. The nanosheets exhibited sustained oxygen generation in both acidic and neutral environments, suggesting their potential to improve local oxygen availability (Fig. 2d). This oxygen-generating activity may support the metabolic shift of immune and stromal cells towards a pro-healing phenotype.

The functional transformation of macrophages is closely related to their metabolic state, and mitochondria are the core of metabolic regulation [48], whereas persistent lactic acidosis in the trauma niche is detrimental to mitochondria, leading to mitochondrial depolarization, reduced ATP production, and impaired pro-repair functions of macrophages [[49], [50], [51]]. Even if macrophages are morphologically induced to an M2-like phenotype, their damaged mitochondria cannot provide sufficient energy to effectively perform their complex pro-repair functions, such as synthesizing and secreting large amounts of growth factors and extracellular matrix. We evaluated the acid-neutralization capacity of CALM. Titration tests showed that the addition of CALM dramatically increased the pH of acidic PBS solutions, demonstrating its excellent acidity-reversing properties (Fig. 2e, Fig. S7b and c). Furthermore, CALM dispersions exhibited a strong buffering capacity, effectively preventing acidification upon the addition of 1% HCl, similar to a standard PBS buffer (Fig. S7a). Functionally, the CALM coating primarily acts as a macroscopic regulator to neutralize acidic media and scavenge extracellular ROS. Additionally, given their approximately 300 nm lateral dimension, it is well-established in the literature that such nanosheets can also be internalized by macrophages [52]. Upon potential cellular uptake, the highly acidic lysosomal compartments would further accelerate the dissolution of the LDH framework. The degradability of CALM was also evaluated in vitro by dispersing the nanosheets in PBS with different pH values or H_2_O_2_ concentrations. As demonstrated in Fig. 2f and g, CALM exhibited stimuli-responsive degradation when dispersed in solutions of varying pH or H_2_O_2_ concentrations (Fig. 2h). SEM images obtained after incubation in solutions with different pH values further showed the degradation behavior of CALM under acidic conditions (Fig. 2i). This microenvironment-responsive biodegradation is primarily driven by the acid-triggered dissolution of the LDH framework, which simultaneously consumes protons and releases the therapeutic ions. To quantitatively monitor this degradation process and the subsequent liberation of functional components, the cumulative release profiles of Ca, Al, and Mn ions were evaluated via ICP-OES in both neutral and acidic media. In the neutral environment, the release of all three metallic ions remained at a low baseline level, indicating the robust structural stability of the coating under physiological conditions. Conversely, exposure to the acidic solution remarkably accelerated the framework dissolution, yielding a sustained and significantly higher cumulative release of Ca^2+^, Al^3+^, and Mn^2+^ (Fig. S8). This quantitative ion-release behavior confirmed that the CALM coating serves as an intelligent, microenvironment-triggered on-demand ion delivery platform. Photographs of CALM nanosheets dispersed in PBS containing different H_2_O_2_ concentrations further showed their ROS-responsive behavior (Fig. 2j). This dual-function behavior makes CALM an intelligent platform for actively remodeling the hostile fracture microenvironment. Furthermore, the stability and release characteristics of the coating upon continuous exposure to physiological fluids were investigated. Following immersion in simulated body fluid (PBS, pH 7.4) for up to 14 days, SEM observations revealed that the coating maintained its structural integrity without macroscopic peeling or detachment (Fig. S9).

### 2.3 In vitro remodeling of mitochondrial homeostasis and mitophagy-related quality control

**Fig. 3:**
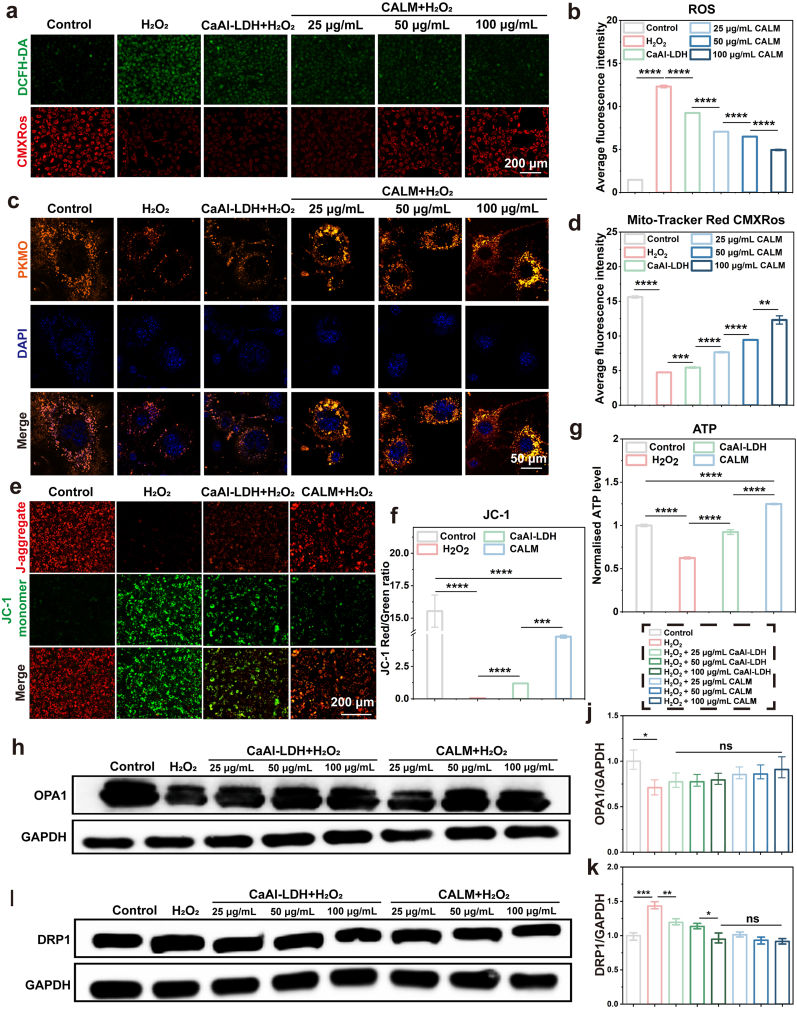
In vitro remodeling of mitochondrial homeostasis. a) Intracellular ROS of RAW264.7 cells labeled by DCFH-DA (green fluorescence) and CMXRos (red fluorescence), and b), d) corresponding fluorescence quantification. c) Representative images of mitochondrial architecture labeled by PKMO (orange fluorescence). e) Representative images of mitochondrial membrane potential detected by JC-1 dye and f) corresponding quantitative analysis. g) Quantitative results of ATP levels in different groups. h-k) Expression levels of mitochondrial fission and fusion-related proteins in different groups. Scale bar: 200 μm (DCFH-DA images and JC-1 images) and 50 μm (PKMO images). Data in (b), (d), (f), (g), (j) and (k) were presented as mean values ± S.D. (n = 3). Data are presented as mean ± SD. Statistical significance was assessed using unpaired two-tailed Student's t-tests or one-way ANOVA followed by Tukey's multiple-comparisons test, as appropriate. Statistical significance was defined as follows: ns, *p >* 0.05; ∗ *p <* 0.05; ∗∗ *p <* 0.01; ∗∗∗ *p <* 0.001; ∗∗∗∗ *p <* 0.0001. (For interpretation of the references to color in this figure legend, the reader is referred to the Web version of this article.)

Encouraged by the excellent ROS-scavenging properties, we further investigated the capacity of CALM to restore mitochondrial homeostasis in RAW264.7 cells under oxidative stress induced by H_2_O_2_ in vitro. First, we investigated the correlation between intracellular ROS levels and mitochondrial health. As anticipated, exposure to 200 μM H_2_O_2_ for 2 h significantly triggered a marked increase in cellular ROS signaling and disrupted the mitochondrial inner membrane. Conversely, CALM treatment significantly reduced ROS levels in a concentration-dependent manner and successfully maintained mitochondrial homeostasis (Fig. 3a, b, d). Correspondingly, fluorescence imaging using a PK Mito Orange probe revealed that CALM protected mitochondrial architecture; cells in the CALM-treated groups displayed well-defined mitochondrial networks with distinct cristae, whereas mitochondria in the H_2_O_2_ group appeared fragmented and structurally unclear (Fig. 3c). This protective effect was further corroborated by Mito Tracker staining (Fig. S10). To obtain a more profound ultrastructural insight into these morphological alterations, transmission electron microscopy (TEM) was employed. Consistent with the fluorescence observations, the mitochondrial cristae in the untreated group exhibited a distinct, typical flat lamellar structure arranged neatly and perpendicularly to the longitudinal axis (Fig. S11). In stark contrast, under pathological stress, the cristae were noticeably diminished, accompanied by partial fragmentation, blurred boundaries, and matrix vacuolization. Strikingly, treatment with CALM remarkably restored the continuity and structural integrity of the mitochondrial cristae, demonstrating a superior reparative efficacy compared to the CaAl-LDH group. A stable mitochondrial membrane potential (MMP) is fundamental to mitochondrial function [53]. We used JC-1 staining to evaluate MMP, where a high red-to-green fluorescence ratio indicates healthy, polarized mitochondria. H_2_O_2_ exposure caused a significant drop in the JC-1 red/green ratio, signifying mitochondrial depolarization. Remarkably, CALM treatment effectively reversed this effect, restoring the ratio to near-normal levels and demonstrating its ability to protect MMP from oxidative damage (Fig. 3e and f).

Mitochondrial dysfunction, a hallmark of aging-related diseases like osteoporosis, leads to impaired ATP synthesis and an accumulation of mitochondrial ROS [54,55], resulting in ATP synthesis reduction and mitochondrial ROS increase, which in turn causes more severe mitochondrial damage. A high NAD+/NADH ratio is essential for fueling ATP production and signifies a healthy metabolic state. We found that CALM treatment effectively attenuated the H_2_O_2_-induced drop in both ATP levels and the NAD+/NADH ratio, indicating that it successfully preserved mitochondrial bioenergetic function under oxidative stress (Fig. 3g, Fig. S12). The balance between mitochondrial fission and fusion is critical for adapting to cellular stress and metabolic demands [56]. By precisely regulating the division and merging of mitochondria, the stability of their quality, quantity and function is maintained, which is the basis for cells to adapt to metabolic demands and stress responses [57,58]. Fission is primarily mediated by Dynamic-related protein 1 (Drp1) [59], while fusion is controlled by proteins like Optic Atrophy Type 1 (Opa1) [60]. Under cellular stress, fission often predominates. Our Western blot analysis revealed that under CALM treatment, the expression of the fission-promoting protein Drp1 was downregulated (Fig. 3h, i, j, k). This shift in the fission/fusion balance towards a less fragmented, more fused mitochondrial network is indicative of improved mitochondrial health and functional optimization.

To further evaluate the biologically relevant hypoxia-modulating activity of the CALM nanocatalyst, we examined the mRNA expression of *Hif1a* and its downstream glycolysis-associated target *Glut1* in RAW264.7 macrophages by qRT-PCR. Under a simulated pathological microenvironment combining physical hypoxia (1% O2) with oxidative stress, the expression levels of Hif1a and Glut1 were markedly upregulated, consistent with activation of a hypoxia-associated glycolytic transcriptional response. CALM treatment significantly reduced the expression of both genes, suggesting that CALM attenuates hypoxia-related transcriptional activation and may help attenuate hypoxia-related metabolic stress in macrophages (Fig. S13).

**Fig. 4:**
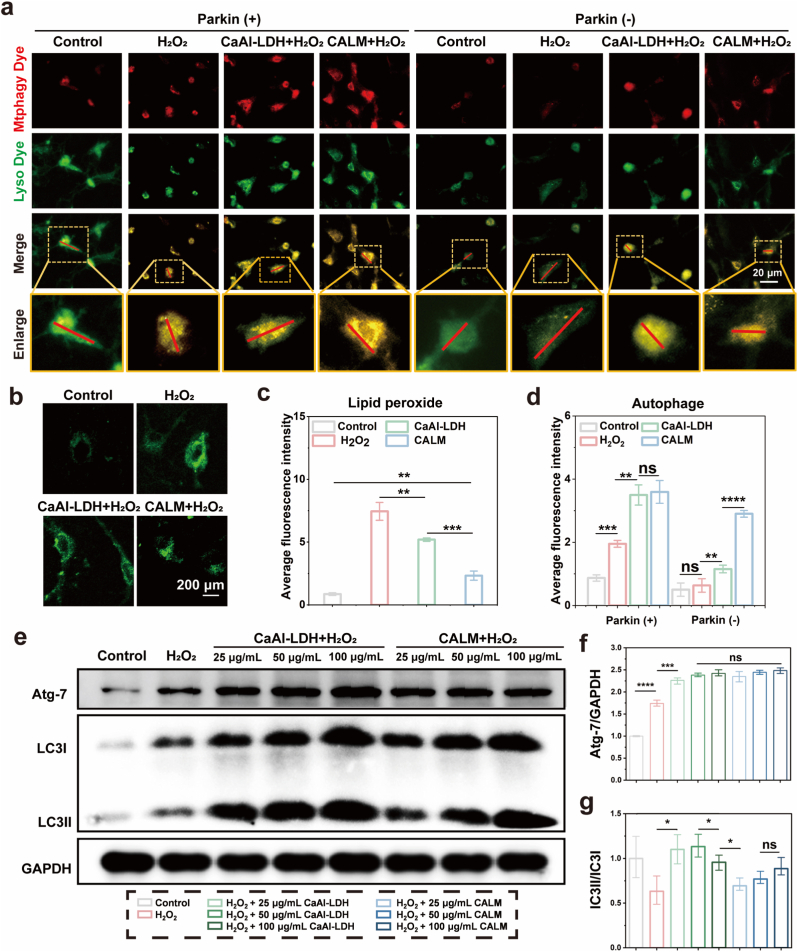
In vitro evaluation of mitophagy-related quality control markers. a) Representative images of mitophagy, and d) corresponding quantitative analysis. b) Representative images of lipid peroxidation, and c) corresponding quantitative analysis. e-g) Expression levels of autophagy-related proteins in different groups. Scale bar: 20 μm (co-staining images) and 200 μm (Lipid peroxide images). Data in (c), (d), (f) and (g) were presented as mean values ± S.D. (n = 3). Data are presented as mean ± SD. Statistical significance was assessed using unpaired two-tailed Student's t-tests or one-way ANOVA followed by Tukey's multiple-comparisons test, as appropriate. Statistical significance was defined as follows: ns, *p >* 0.05; ∗ *p <* 0.05; ∗∗ *p <* 0.01; ∗∗∗ *p <* 0.001; ∗∗∗∗ *p <* 0.0001.

Mitophagy is a crucial quality control mechanism within cells, which specifically eliminates damaged or redundant mitochondria through the autophagy process. When mitophagy is incomplete, dysfunctional mitochondria will accumulate and trigger a series of chain reactions including aggravating oxidative stress and hindering M2 polarization [61,62]. Further, accumulated ROS drives lipid peroxidation [63,64]. The products of lipid peroxidation, such as malondialdehyde -MDA, can directly damage the function of bone-forming cells, while oxidatively modified lipids can amplify inflammatory signals [65]. In the context of fracture healing, intensified lipid peroxidation may deplete the osteocytes required for repair by inducing ferroptosis [66]. Therefore, we investigated whether CALM influences mitochondrial quality control via mitophagy. Using co-staining with mitophagy and lysosome dyes in Parkin-overexpressing cells, we observed that exposure to H_2_O_2_ significantly increased autophagosome formation, reflecting an upregulation of mitophagy in response to oxidative stress, while treatment with CALM significantly further enhanced the colocalization of mitochondria with lysosomes, suggesting increased mitochondrial-lysosomal colocalization under oxidative stress (Fig. 4a–d, Fig. S14). Western blot analysis further confirmed this, showing increased expression of the key mitophagy-related proteins Atg-7, an essential E1-like activating enzyme required for the initial steps of autophagosome biogenesis, while LC3-II/LC3-I ratio did not exhibit a corresponding significant increase (Fig. 4e, f, g). LC3-II, a key marker of autophagic vesicles, facilitates autophagosome formation and subsequent lysosomal degradation of mitochondria [67]. Together with the mitochondrial-lysosomal colocalization data, these marker changes suggest that CALM is associated with mitophagy-related mitochondrial quality control under oxidative stress. We observed a significant reduction in lipid peroxidation, a process driven by ROS that can induce ferroptosis and amplify inflammation, in CALM-treated cells (Fig. 4b and c). Collectively, these findings suggest that CALM helps preserve mitochondrial homeostasis by scavenging ROS, maintaining mitochondrial integrity and function, rebalancing mitochondrial dynamics, and modulating cellular quality-control mechanisms.

### 2.4 In vitro immunomodulation and macrophages reprogramming

**Fig. 5:**
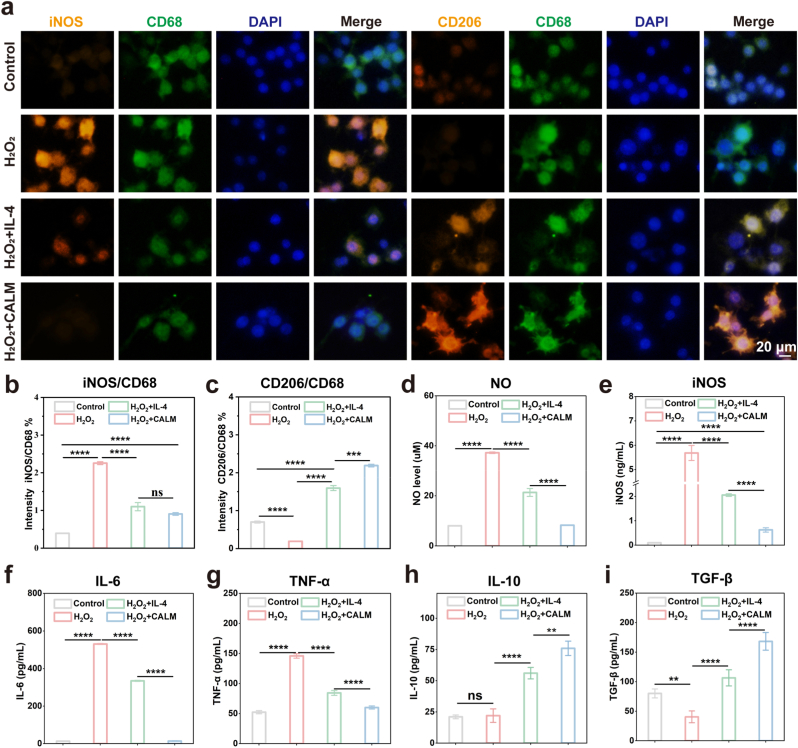
In vitro immunomodulation and macrophages reprogramming. a) Representative images of immunofluorescence staining on macrophage polarization marker, and b-c) corresponding quantitative analysis. d-i) Quantitative results of inflammatory mediators and key pro-inflammatory and anti-inflammatory cytokines in different groups. Scale bar: 20 μm. Data in (b), (c), (d), (e), (f), (g), (h) and (i) were presented as mean values ± S.D. (n = 3). Data are presented as mean ± SD. Statistical significance was assessed using unpaired two-tailed Student's t-tests or one-way ANOVA followed by Tukey's multiple-comparisons test, as appropriate. Statistical significance was defined as follows: ns, *p >* 0.05; ∗ *p <* 0.05; ∗∗ *p <* 0.01; ∗∗∗ *p <* 0.001; ∗∗∗∗ *p <* 0.0001.

Macrophages exhibit remarkable plasticity, and their phenotypic transition from a pro-inflammatory M1 state to a pro-reparative M2 state is considered a critical molecular switch for successful fracture healing [68]. A timely M1-to-M2 conversion is essential for recruiting BMSCs and orchestrating subsequent regenerative responses [69]. Therefore, we investigated whether CALM could effectively drive this beneficial phenotypic shift in macrophages. To assess its immunomodulatory potential, we first induced a classic M1 inflammatory phenotype in RAW264.7 cells using H_2_O_2_. We then evaluated the ability of CALM to counteract this pro-inflammatory polarization. Immunofluorescence staining revealed that co-treatment with CALM significantly reduced the percentage of iNOS-positive (M1 marker) cells compared to the H_2_O_2_-stimulated group. Concurrently, the percentage of CD206-positive (M2 marker) cells was markedly elevated in the presence of CALM, indicating a robust promotion of the M2 phenotype (Fig. 5a, b, c). To quantitatively corroborate the immunofluorescence findings, the transcriptional profiles of classical macrophage polarization markers were evaluated via quantitative real-time PCR (qRT-PCR). As shown in Fig. S15, H_2_O_2_ exposure significantly upregulated the mRNA levels of M1-associated pro-inflammatory genes (*Nos2* and *TNF-α*), confirming the ROS-induced pro-inflammatory state. Conversely, treatment with CALM profoundly suppressed the expression of these M1 markers down to near-baseline levels. CALM treatment remarkably upregulated the expression of M2-associated pro-reparative genes (*Arg-1* and *CD206*), and the transcriptional activation of *Arg-1* and *CD206* in the CALM group was comparable to that of the IL-4 positive control group. Consistent with these cellular-level changes, the secretion of M1-associated inflammatory mediators was also substantially suppressed. Compared to the H_2_O_2_ group, the CALM-treated group exhibited significantly lower levels of nitric oxide (NO), the product of iNOS activity (Fig. 5d and e). Furthermore, ELISA results confirmed that CALM treatment effectively downregulated the expression of the key pro-inflammatory cytokines TNF-α and IL-6 (Fig. 5f and g) and upregulated the expression of anti-inflammatory cytokines IL-10 and TGF-β (Fig. 5h and i). Collectively, our findings demonstrate that CALM possesses potent immunomodulatory properties, capable of inhibiting H_2_O_2_-induced M1 macrophage polarization while simultaneously promoting a switch towards the M2 phenotype. This effect is likely underpinned by CALM's intrinsic ability to suppress intracellular oxidative stress, a key driver of M1 activation, thereby creating a favorable immune microenvironment for tissue regeneration. The immunomodulatory effect of the CALM coating is primarily driven by the synergistic actions of the Ca^2+^/c-Maf axis and the PI3K/Akt axis. As the CaAl-LDH framework dissolves in the acidic fracture microenvironment, localized release of calcium ions and the *in situ* formation of calcium phosphate nanoparticles occur. These entities can be recognized by the calcium-sensing receptor (CaSR) on macrophages, triggering intracellular calcium oscillations that subsequently activate the c-Maf transcription factor, which is a critical driver for M2 macrophage polarization [70,71]. Simultaneously, the ROS-scavenging capability of MnOx helps preserve mitochondrial homeostasis and activates the PI3K/Akt signaling pathway. Together, the Ca^2+^/c-Maf and PI3K/Akt axes orchestrate a comprehensive network that strongly promotes a pro-regenerative niche.

### 2.5 In vitro osteogenic activity

**Fig. 6:**
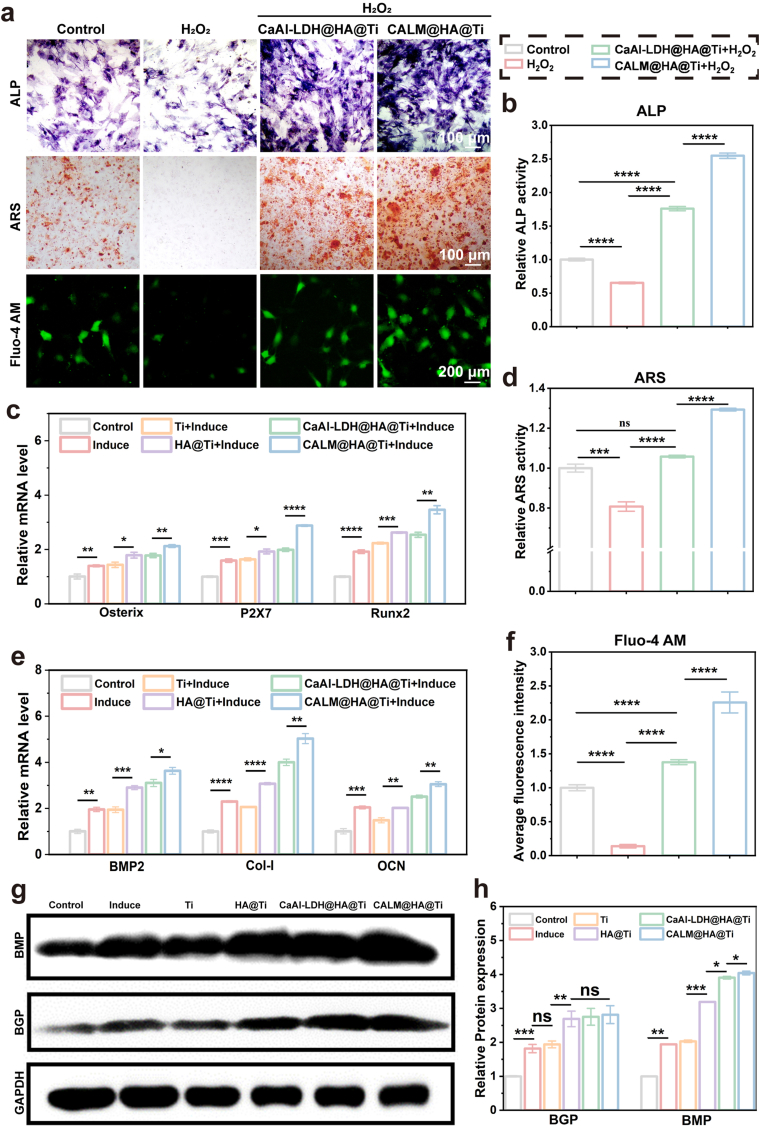
In vitro osteogenic activity. a) Representative images of ALP staining, ARS staining, and intracellular Ca^2+^, and b), d), f) corresponding quantitative analysis. c) and e) Osteogenic differentiation related genes expression of BMSCs in different groups. g-h) Osteogenic differentiation related proteins expression of BMSCs in different groups. Scale bar: 100 μm (ALP and ARS images) and 200 μm (Fluo-4AM images). Data in (b), (c), (d), (e), (f) and (h) were presented as mean values ± S.D. (n = 3). Statistical significance was assessed using unpaired two-tailed Student's t-tests or one-way ANOVA followed by Tukey's multiple-comparisons test, as appropriate. Statistical significance was defined as follows: ns, *p >* 0.05; ∗ *p <* 0.05; ∗∗ *p <* 0.01; ∗∗∗ *p <* 0.001; ∗∗∗∗ *p <* 0.0001.

Encouraged by the demonstrated microenvironment-modulating and immunomodulatory capabilities of the CALM coating, we proceeded to evaluate its direct impact on the osteogenic differentiation of BMSCs in vitro. First of all, biocompatibility evaluation is essential for implantable materials. The CCK-8 assay showed no significant differences in absorbance among the groups after 3 h of culture (Fig. S16a). To further evaluate the extended cytocompatibility of the modified coatings, the CCK-8 assay was extended to 1, 4, and 7 days of culture. There was no statistically significant difference in the relative cell viability among the control, CaAl-LDH, and CALM groups at any predetermined time point (Fig. S16b). These results indicate favorable in vitro cytocompatibility of CALM under the tested conditions. Alkaline phosphatase (ALP), a key marker of early osteogenic differentiation, facilitates bone mineralization by hydrolyzing phosphate esters [72]. To simulate the oxidative stress conditions of a fracture microenvironment, BMSCs were cultured on the various substrates in the presence of H_2_O_2_. On day 7, both qualitative staining and quantitative analysis revealed the highest level of ALP activity in the CALM@HA@Ti + H_2_O_2_ group, suggesting that the CALM coating effectively protects BMSCs from oxidative stress and promotes their differentiation (Fig. 6a and b). We then evaluated matrix mineralization, a hallmark of late-stage osteogenesis, via Alizarin Red staining. Consistent with the ALP results, the formation of mineralized calcium nodules was most pronounced in the CALM@HA@Ti + H_2_O_2_ group (Fig. 6a–d). We hypothesized that the release of calcium ions from the degrading CALM coating plays a pivotal role. Ca^2+^ is indispensable for osteoblast differentiation and biomineralization. Using the fluorescent probe Fluo-4 AM, we observed a significant intracellular Ca^2+^ influx in BMSCs exposed to CALM, indicating the activation of calcium-dependent signaling pathways (Fig. 6a–f). Beyond direct signaling, the degradation of the CaAl-LDH component releases Ca^2+^ that can react with phosphate in the body fluid to form calcium phosphate (CAP) precipitates *in situ*. This process is doubly beneficial: not only does it contribute to local biomineralization, but CAPs are also known to induce M2 macrophage polarization [73,74]. This creates a positive feedback loop where the material degradation product enhances the pro-regenerative immune microenvironment, which in turn further promotes osteogenesis. To verify whether the CALM-reprogrammed immune microenvironment could actively rescue and augment bone regeneration, a macrophage-conditioned medium (CM) osteogenic induction assay was performed on BMSCs. Consistent with the ALP and ARS staining results, the CM assay further indicated that CALM-conditioned macrophages created a more pro-osteogenic microenvironment for BMSCs (Fig. S17).

To elucidate the underlying molecular mechanisms, we analyzed the expression of key osteogenic markers at the gene and protein levels. On day 7, qRT-PCR analysis showed that BMSCs cultured on CALM@HA@Ti exhibited significantly upregulated expression of crucial osteogenic transcription factors (*Runx2, Osterix*) and bone-related genes, including *P2X7, BMP-2, Col-I, and OCN* (Fig. 6c–e). These findings at the transcript level were further corroborated by Western blot analysis, which confirmed a marked increase in the protein expression of BMP and BGP (Fig. 6g and h). It can be speculated that microenvironment improvement and immunomodulation of CALM is favorable for cell differentiation into the osteoblast phenotype, which may accelerate bone mineralization through enhanced calcium deposition [75]. Therefore, the osteogenic potential of CALM is a synergistic effect of direct cellular stimulation, sustained ion release, and the contribution of the underlying HA substrate [76].

### 2.6 In vivo promoting osteoporotic bone fracture healing effect

**Fig. 7:**
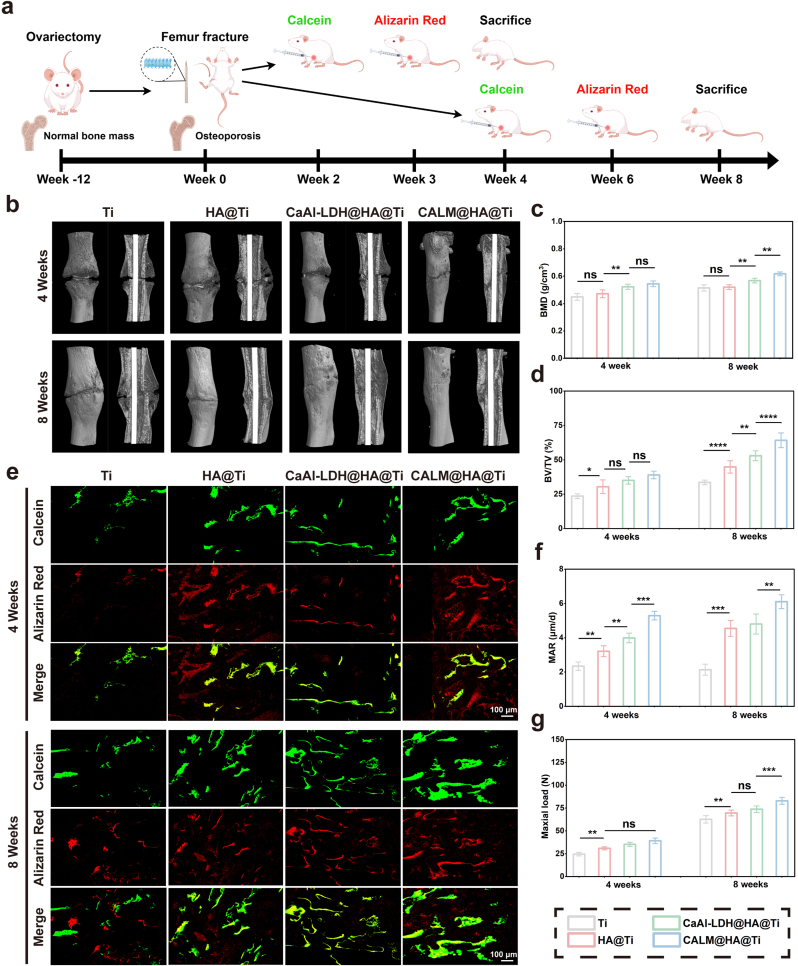
In vivo promoting osteoporotic bone fracture healing effect. a) Schematic illustration of the osteoporotic rat femoral fracture model and treatment procedure. b) Representative micro-CT images of fractured femurs at 4 and 8 weeks post-surgery across different groups. c-d) Quantitative analysis of BMD and BV/TV of the newly formed bone based on micro-CT data. e) Representative images of sequential fluorescence labeling for bone regeneration, where green (calcein) and red (Alizarin Red S) indicate newly formed mineralized tissue at different time points. f) MAR in rats at 4 and 8 weeks post-surgery. g) Biomechanical evaluation of the maximal load at the fracture site. All in vivo experiments were conducted with six biological replicates (n = 6). Statistical significance was assessed using unpaired two-tailed Student's t-tests or one-way ANOVA followed by Tukey's multiple-comparisons test, as appropriate. Statistical significance was defined as follows: ns, p > 0.05; ∗ p < 0.05; ∗∗ p < 0.01; ∗∗∗ p < 0.001; ∗∗∗∗ p < 0.0001. (For interpretation of the references to color in this figure legend, the reader is referred to the Web version of this article.)

While the CALM nanocomposite demonstrated excellent pro-osteogenic properties in vitro, its therapeutic efficacy in a complex physiological environment required rigorous in vivo validation. To this end, we investigated the performance of CALM@HA@Ti implants in promoting fracture healing within a clinically relevant osteoporotic rat model. These engineered implants were designed to serve a dual function: providing immediate biomechanical stability to the fracture site and acting as a local delivery platform for the CALM nanocomposite. The overall experimental design is illustrated in Fig. 7a. An osteoporotic rat model was first established, characterized by significantly reduced bone volume/total volume (BV/TV) and trabecular thickness (Tb.Th) compared to healthy rats (Fig. S18). Subsequently, a linear femur fracture was created in these animals, and different bare Ti-based rods (Ti, HA@Ti, CaAl-LDH@HA@Ti, and CALM@HA@Ti) were implanted into the femoral epiphysis (Fig. S19). All animals recovered well post-surgery without complications. To ensure the integrity of the coatings during implantation, the medullary cavity was pre-dilated, and post-operative analysis confirmed that coating loss was minimal, ensuring the reliability of our findings.

Fracture healing was monitored at 4 and 8 weeks post-surgery using micro-computed tomography (micro-CT). Qualitative 3D reconstructions revealed substantially more mature and bridged callus formation in the CALM@HA@Ti group compared to the control groups at both time points (Fig. 7b). This visual observation was substantiated by quantitative morphometric analysis of the region of interest (ROI). The CALM@HA@Ti group exhibited significantly higher bone mineral density (BMD) and BV/TV ratios at both 4 and 8 weeks , indicating accelerated and superior bone regeneration (Fig. 7c and d). To assess the quality and rate of new bone formation, we employed polyfluorochrome sequential labeling. The mineral apposition rate (MAR), which measures the speed of new bone deposition, was calculated from the distance between calcein and alizarin red fluorescent labels. The CALM@HA@Ti group displayed a significantly higher MAR than both control groups at 4 and 8 weeks, signifying a more rapid and robust osteogenic response at the implant interface (Fig. 7e and f). Histological analysis via H&E and Masson's trichrome staining further corroborated these findings, revealing abundant, well-organized new bone and mature collagen matrices in the peri-implant region of the CALM@HA@Ti group (Fig. S20). CALM-treated tissues did not exhibit obvious local inflammatory cell infiltration compared to the uncoated Ti group. To directly confirm whether the accelerated bone regeneration in vivo was driven by the hypothesized immunometabolic reprogramming, we evaluated the macrophage polarization state directly within the local fracture callus via immunofluorescence staining at 4-week post-surgery. In the CALM-coated group, iNOS expression was reduced, whereas CD206 expression was enriched at the peri-implant callus site. Quantitative fluorescence analysis showed the same trend (Fig. S21). These *in situ* immunostaining results support an association between CALM coating and a shift toward a pro-reparative macrophage phenotype in vivo. Ultimately, the goal of fracture treatment is the restoration of mechanical function. Biomechanical testing of the healed femurs demonstrated that the CALM@HA@Ti group could withstand the highest maximal bending load, correlating directly with the superior healing observed radiographically and histologically (Fig. 7g). Furthermore, short-term systemic biosafety-related indicators were evaluated. Analysis of liver and kidney function indicators at 2 and 4 weeks post-surgery showed no significant differences between the treated and untreated rats, suggesting no apparent short-term hepatic or renal toxicity under the present experimental conditions (Fig. S22). Although the present study provided preliminary evidence of controlled Ca/Mn/Al release, short-term liver and kidney function, local inflammatory response, and coating stability during implantation, comprehensive long-term biosafety remains to be established. In particular, future studies should systematically evaluate Mn/Al biodistribution and clearance, possible metal ion accumulation in major organs, long-term organ histopathology, hemocompatibility, and chronic degradation behavior before clinical translation. The mechanical durability and interfacial bonding of the composite coating are critical for ensuring sustained therapeutic efficacy post-implantation. To evaluate coating integrity during surgical insertion, attrition rates were quantified. The results showed low material loss, with attrition rates of 3.85% ± 1.97% for the CALM@HA@Ti group, 4.09% ± 1.74% for the CaAl-LDH@HA@Ti group, and 3.48% ± 1.01% for the HA@Ti group (Fig. S23). This low degree of physical detachment quantitatively supports the mechanical retention of the coating during initial surgical insertion, which is consistent with strong electrostatic interactions between the CaAl-LDH framework and the titanium substrate. Despite the promising in vivo performance of the CALM coating, long-term mechanical stability remains a potential limitation. While our electrostatic adsorption strategy minimizes coating attrition during initial surgical insertion, orthopedic implants are continuously subjected to complex physiological shear forces during long-term weight-bearing activities. Over time, physical detachment of the nanosheets could occur. Future translational research should focus on strengthening interfacial bonding, for example through covalent coupling, mineral interlocking, or polymer-assisted adhesive layers, to improve long-term mechanical resilience.

### 2.7 Absolute quantitative transcriptome sequencing analysis

**Fig. 8:**
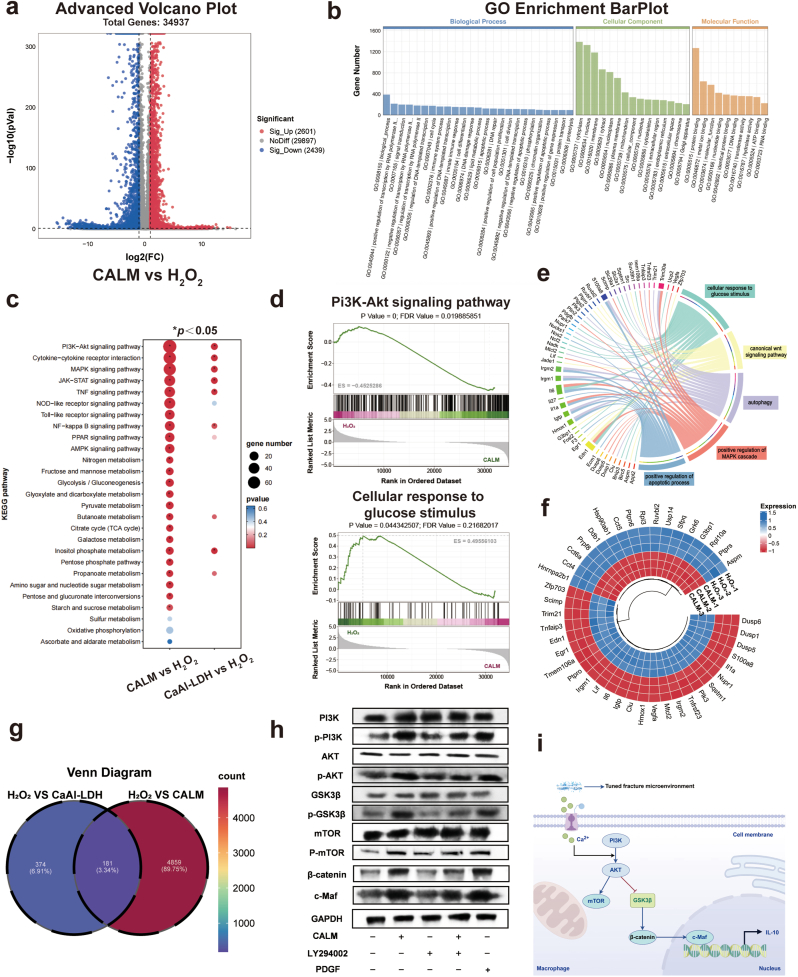
Transcriptome and mechanistic analysis of osteogenic induction mediated by CALM. a) Volcano plot displaying 2601 upregulated and 2439 downregulated genes between the H_2_O_2_ group and the CALM group. b) GO enrichment analysis of DEGs. c) Bubble chart based on KEGG pathway enrichment analysis of DEGs. d) GSEA enrichment analysis. e) Chord diagram illustrating shared DEGs involved in multiple signaling pathways. f) Heatmap showing the differential expression patterns of DEGs; blue represents upregulated genes, and red indicates downregulated genes. g) Venn plot of mRNA changes in different groups. h) Western blot analysis of related proteins, including PI3K, p-PI3K, AKT, p-AKT, GSK3β, p-GSK3β, mTOR, p-mTOR. LY294002 and PDGF were used as a PI3K-Akt pathway inhibitor and activator, respectively. i) Schematic mechanism of CALM-mediated oxidative-stress regulation, immunomodulation, and macrophage reprogramming. (For interpretation of the references to color in this figure legend, the reader is referred to the Web version of this article.)

To elucidate the molecular mechanisms by which CALM modulates macrophage function under the oxidative stress characteristic of a fracture site, we conducted a high-throughput transcriptome sequencing analysis on RAW264.7 macrophages to compare the differentially expressed genes (DEGs) between treatment with control, H_2_O_2_, CaAl-LDH and CALM. Principal component analysis (PCA) and Pearson correlation heatmaps confirmed that the CALM-treated group had a distinct gene expression profile, indicating a profound transcriptomic impact (Fig. S24a). Volcano plot analysis revealed 2601 significantly upregulated and 2439 significantly downregulated genes in the CALM group compared to the H_2_O_2_-treated group (Fig. 8a). Changes in gene expression in the other group comparisons are shown in Fig. S24b and c. These transcriptional differences confirmed that the oxidative-stress model and material treatments produced distinct cellular responses.

To understand the biological functions of these DEGs, we performed Gene Ontology (GO) and KEGG pathway analyses. GO analysis reveals the changes of these genes in biological processes, cellular components, and molecular functions (Fig. 8b). Notably, a bubble chart based on KEGG analysis identifies that DEGs were predominantly enriched in multiple critical immunomodulatory pathways, including the PI3K-Akt signaling pathway, MAPK signaling pathway, JAK-STAT signaling pathway, TNF signaling pathway, Toll-like receptor signaling pathway (Fig. 8c). This suggests that therapeutic effects of CALM are mediated through a coordinated regulation of these central inflammatory hubs. As demonstrated in our prior studies [77], the PI3K-Akt signaling pathway, a known pivotal regulator of osteogenesis and immune cell function, emerged as a central hub warranting deeper investigation.

Moreover, Gene set enrichment analysis (GSEA) further underscored the central role of the PI3K-Akt signaling pathway, which was significantly enriched in the CALM-treated group compared to the H_2_O_2_ group (Fig. 8d, Fig. S24d). The activation of this pathway is a critical switch that promotes the anti-inflammatory M2 macrophage phenotype while suppressing the pro-inflammatory M1 state [78,79]. Activated PI3K-Akt signaling is known to inhibit NF-κB activity, thereby reducing the secretion of pro-inflammatory cytokines, while also promoting the production of the anti-inflammatory cytokine IL-10 via inhibition of GSK3β [80,81]. Furthermore, the PI3K-Akt-Nrf2 axis is crucial for cellular antioxidant defense. Experiments have demonstrated that blocking the PI3K-Akt pathway with pharmacological inhibitors significantly weakens the expression of Nrf2 target genes in macrophages, making the cells more prone to damage and inflammatory responses under oxidative stress conditions [82]. Therefore, the upregulation of PI3K-Akt signaling pathway may play a pivotal role in the CALM-mediated regulation of macrophage polarization and antioxidant/anti-inflammatory responses, which may sequentially activate mTOR and the downstream β-catenin by inhibiting GSK3β. The chord diagram further demonstrated the complex relationships between DEGs and enriched signaling pathways, providing a more comprehensive understanding of the molecular mechanisms underlying the macrophage polarization and antioxidant by CALM (Fig. 8e). It is well-known that the MAPK signaling pathway, Toll-like receptor signaling pathway, and NF-κB signaling pathway significantly amplify local inflammatory responses, promote the secretion of pro-inflammatory cytokines, and its cascade reaction is the core connection point between inflammatory signaling and metabolic reprogramming [[83], [84], [85]]. Concurrently, we observed significant downregulation of DEGs within other pro-inflammatory cascades, such as the MAPK and Toll-like receptor pathways (e.g., Ruvbl2, Sfpq, Grk6), and upregulation of anti-inflammatory regulators (e.g., Tnfaip3, Hmox1, Dusp1/5/6). Circular plot further confirms that the above inflammatory signaling pathways are closely linked to inflammation-related genes, indicating a strong antioxidant/anti-inflammatory effect induced by CALM (Fig. 8f). This coordinated regulation suggests that CALM actively suppresses inflammatory hubs while promoting pro-regenerative signaling. Furthermore, as shown in Fig. 8g, the intersection between two groups in a Venn plot presented that 181 upregulated mRNAs were uniquely associated with the incorporation of MnOx in CALM, suggesting that MnOx incorporation contributes to part of the therapeutic transcriptional signature.

Based on this comprehensive transcriptomic evidence, we hypothesized that CALM exerts its therapeutic effects primarily through the activation of the PI3K/Akt/GSK3β/β-catenin signaling cascade. To validate this hypothesis, we performed Western blot analysis to quantify the expression of key proteins in this pathway. As predicted, the phosphorylation levels of PI3K, Akt, and GSK3β were all significantly elevated in the CALM group. Specifically, quantitative densitometry revealed that CALM treatment increased the p-PI3K/PI3K ratio by approximately 1.7-fold and the downstream β-catenin expression by 2.0-fold compared to the control group. Furthermore, the addition of the PI3K inhibitor LY294002 significantly suppressed these activations; however, co-treatment with CALM partially rescued the pathway, increasing the p-PI3K/PI3K ratio by roughly 1.4-fold and β-catenin by 1.4-fold relative to the LY294002-only group, thereby supporting the involvement of this pathway. We also observed a significant increase in the p-mTOR/mTOR ratio, a downstream target of AKT (Fig. 8h, Fig. S24e–j). PI3K phosphorylation typically activates Akt, which subsequently phosphorylates and inactivates GSK3β. The inactivation of GSK3β prevents the degradation of β-catenin, allowing its accumulation and nuclear translocation, which is crucial for driving the transcription of M2-associated genes. As demonstrated, LY294002 effectively suppressed p-Akt, relieved the inhibitory phosphorylation of GSK3β, and consequently led to the degradation of β-catenin, thereby arresting the M1-to-M2 polarization. Conversely, CALM or PDGF treatment reversed this cascade, supporting that CALM may exert part of its immunomodulatory effect precisely through the PI3K/AKT/GSK3β/β-catenin axis [86]. While we focused on the PI3K/Akt pathway, the enrichment of the MAPK and JAK-STAT pathways is also significant. It is likely that CALM exerts its potent immunomodulatory effects not through a single pathway, but by coordinating a systems-level response across these major signaling hubs. In summary, our transcriptomic analysis combined with functional validation provides mechanistic evidence suggesting that CALM remodels mitochondrial homeostasis and orchestrates a pro-regenerative cellular response by activating the PI3K/AKT/GSK3β/β-catenin signaling pathway. This activation governs immunomodulation and macrophage reprogramming, which in turn protects BMSCs from oxidative stress and robustly promotes their osteogenic differentiation, ultimately leading to enhanced bone regeneration (Fig. 8i).

## 3 Conclusion

In this study, we have developed a dual-responsive CALM nanocatalytic medicine designed to modulate the hostile microenvironment of osteoporotic fractures. By scavenging ROS, generating oxygen, and buffering acidosis, the CALM helps preserve mitochondrial homeostasis and promotes the metabolic and phenotypic reprogramming of macrophages toward a pro-regenerative M2 state, thereby enhancing osteoporotic fracture healing. Mechanistically, transcriptomic and protein-level analyses indicate that the PI3K/Akt/GSK3β signaling pathway contributes to CALM-mediated immunomodulation and bone repair. Overall, CALM provides a promising nanocatalytic coating strategy for osteoporotic fracture healing.

## 4 Methods

### 4.1 Plasma spraying

Titanium alloy Kirschner wires (1.0 mm in diameter and 30 mm in length, produced by Jiangsu Runhui Medical Technology Co., Ltd., China) were used as substrates for the in vivo experiment. The wires were cleaned in deionized water, followed by air drying. HA powder with particle size ranged from 15 to 30 μm was applied by using an atmospheric plasma system (Medicoat AG, Switzerland), as reported previously [87,88].

### 4.2 Synthesis of 2D CALM nanosheets

CaAl-LDHs have been synthesized by dissolving 32 mmol of Ca (NO_3_)_2_·4H_2_O and 16 mmol of Al (NO_3_)_3_·9H_2_O in 250 mL of decarbonated water with constant stirring under a stream of nitrogen (XL grade, 99.99% pure) gas. A white gelatinous suspension was obtained and aged for 24 h at room temperature under continuous nitrogen purging. The suspension was then collected and centrifuged to separate the precipitate followed by washing several times with decarbonated water and finally freeze dried to get free flowing nanocrystalline pristine LDH powder [39,41]. CaAl-LDH (0.03 g) was added to 30 mL ultrapure water and ultrasonically dispersed. Then 0.06g MnCl_2_·4H_2_O were added, mixed for 1 h, 0.08 g KMnO_4_ and 0.08 g urea were added, and continued to mix in a water bath pot at 30°C for 5h, finally, after centrifugation, water washing and alcohol washing many times until the supernatant liquid is transparent and colorless. CALM was obtained after centrifugation, washing with water and ethanol, and drying.

### 4.3 Preparation of CALM@HA composite coating

HA-coated wires (HA@Ti) were aminated by immersing in APTES for 1 h, followed by nitrogen drying. CALM nanoparticles were ultrasonically dispersed in PBS (1 mg mL−1). HA@Ti was immersed in the solution, and then volatilized and dried to obtain CALM@HA composite coating. High-temperature sterilization for further use (120°C, 1 h).

### 4.4 Characterization

The morphological characteristics of the nanoparticles and their elemental distribution were examined using scanning electron microscopy (SEM, FEI Quanta 650, Thermo Fisher Scientific, USA). Elemental mapping was performed with energy-dispersive spectroscopy (EDS, Bruker X-Flash 6130, Bruker, Germany). The thickness of the nanosheets was measured using atomic force microscopy (AFM, Bruker Dimension Icon, Bruker Corporation, Germany). The zeta potential of the nanoparticles in phosphate-buffered saline (PBS) was measured using a particle size potential analyzer (BeNano 180 Zeta Max, Bettersize Instruments Ltd, China) to assess their charge. The nanometer particle size in ethanol solution is also measured by the above instruments. X-ray photoelectron spectroscopy (XPS) spectrum was obtained using the K-Alpha (ESCALAB 250XL, Thermo Fisher Scientific, USA) to analyze the chemical composition and bonding states. The surface morphology of the K-wire was measured by scanning electron microscopy (SEM, ZEISS GeminiSEM 300, Carl Zeiss AG, Germany). The XRD patterns were acquired on a Rigaku D/MAX-2250 V diffractometer (40 kV, 40 mA) with a Cu Ka radiation target.

### 4.5 Acid neutralization

Briefly, PBS solutions (pH 4.5 or 6.5) were titrated with CALM (25 mg/mL), and NaOH (0.01 or 0.1 M) solutions (10 μL per titration) with magnetic stirring. The pH values were continuously monitored by a pH meter after each titration (PHB-1, SANXIN, China). The titration was stopped when the titrated solutions experienced three consecutive pH decreases of less than 0.01. Moreover, NaHCO3 (1 M), PBS (pH 7.4), deionized water, and CALM (25 mg/mL) were used as comparison groups and titrated with 1% hydrochloric acid (10 μL per titration) under magnetic stirring. The procedures were similar to those described above.

### 4.6 In vitro degradation capacity

Observation of the solubility of CALM in hydrogen peroxide: CALM (200 μL, 2 mg/mL) was reacted with 200 μL of H_2_O_2_ (1.0, 0.5, 0.25, 0.05, 0.01, and 0.005 mol/L) respectively. The mixture was then observed at predetermined time points: (10, 20 and 30 min). To further investigate solubility, we used a UV spectrophotometer (UV-3600, Shimadzu, Japan) to measure the absorbance of CALM (2 mg/mL) in phosphate-buffered saline (PBS) at different pH values (pH 6, 7, and 8) after dissolving it for 30 min. Additionally, we dissolved CALM (200 μL, 2 mg/mL) in hydrogen peroxide solutions of varying concentrations (0.05, 0.01 and 0.005 mol/L). Then, we centrifuged the solution and measured the absorbance of the supernatant.

PBS solutions adjusted to three different pH values were utilized as the release media. Briefly, the CALM powder was dispersed in the respective PBS to formulate a 5 mg/mL suspension. The suspension was subsequently transferred into a dialysis bag (Molecular Weight Cut-Off: 8000 Da) and tightly sealed. The dialysis bag was then completely submerged in a 50 mL centrifuge tube containing 50 mL of the corresponding release medium, ensuring identical medium composition inside and outside the bag. To simulate the in vivo physiological environment, the centrifuge tubes were incubated in a constant-temperature shaking incubator at 37°C. At predetermined time points (4, 8, 24, 48, and 72 h), 2 mL of the external release medium was accurately withdrawn and immediately replaced with an equal volume of fresh corresponding PBS to maintain a constant volume. The collected samples were centrifuged, and the supernatants were analyzed using ICP-OES (Thermo Fisher Scientific, USA) to quantify the cumulative release concentrations of the relevant ions.

To evaluate the long-term structural stability and degradation behavior of the hierarchical coating under physiological conditions, the CALM-coated substrates were immersed in PBS, (pH 7.4) at 37°C for 14 days. The substrates were then gently rinsed with ultrapure water and dried under nitrogen. The surface morphology of the immersed samples were characterized via SEM.

### 4.7 In vitro biocompatibility evaluation

The biocompatibility of different materials (CaAl-LDH and CALM) toward BMSCs was evaluated using the CCK-8 assay. At predetermined time points (0, 1, 2, and 3h), 10 μL of CCK-8 reagent (Dojindo Lab oratories, Kumamoto, Japan) was added to each well. The plates were incubated for 3 h at 37°C under standard culture conditions. On days 1, 4, and 7 post-seeding, the culture medium was removed, and 400 μL of fresh complete medium containing 10% (v/v) CCK-8 solution was added to each well. After incubation at 37°C for another 2 h, 100 μL of the supernatant from each well was transferred to a 96-well plate. The optical density (OD) value of each specimen was subsequently measured at a wavelength of 450 nm. The relative cell viability was calculated and normalized against the control group.

### 4.8 In vitro production of oxygen and antioxidant capacity

Oxygen generation is monitored using an oxygen generator. Immerse the probe of the oxygen release indicator below the surface of the calm solution and observe the oxygen generation readings. The antioxidant capacity of CALM was evaluated at different pH levels (pH 4.5 or 6.5) using three representative ROS targets: hydrogen peroxide (H_2_O_2_), superoxide anion radical (O_2_·^-^), and total antioxidant capacity. Firstly, the H_2_O_2_ scavenging activity of CALM was evaluated using a molybdic acid method. The reaction mixture was incubated for 10 min, and the absorbance was measured at 405 nm to determine the scavenging efficiency. Additionally, the O_2_·^-^ scavenging activity was evaluated using an SOD activity assay kit (Dojindo, Japan) with the WST-1 method. Finally,the total free radical's capacity of CALM nanosheet was investigated by the antioxidant capacity assay kit (Beyotime Technology Inc., China) with a rapid 2, 2′-azinobis (3-ethylbenzothiazoline 6-sulfonate) (ABTS) method. To further evaluate the intracellular ROS scavenging capacity of the nanocomposite, DCFH-DA (1:1000; S0033, Beyotime Biotechnology, China) was used as a fluorescent marker to detect intracellular ROS levels. Furthermore, Cells (RAW246.7) were incubated with CALM for 12 h, then seeded into a serum-free medium containing a DCFH-DA solution (10 mM). The cells were cultured at 37°C for 30 min. Cells were washed twice with serum-free medium or PBS to thoroughly remove unincorporated DCFH-DA. After centrifugation to remove supernatant, cells were resuspended in PBS for subsequent assays. Finally, cells were fixed with 1% paraformaldehyde. Fluorescence measurements were performed and observed under a microscope at the optimal excitation wavelengths of 488 nm or 525 nm. Additionally, we used the MitoPeDPP kit (Dojindo, Japan) to visualize lipid peroxides on the inner mitochondrial membrane. Lipid peroxides were detected in live cells using fluorescence microscopy after processing with the kit.

### 4.9 In vitro enhancement of macrophage mitochondrial capacity

Raw264.7 cells were cultured in high-glucose Dulbecco's Modified Eagle's Medium (DMEM) supplemented with 10% (v/v) fetal bovine serum (FBS, Gibco) at 37°C in a 5% CO_2_ atmosphere. To simulate the oxidative stress environment in vitro, the culture medium was replaced with medium containing 200 μM H_2_O_2_ for 2 h [89,90]. Following the treatment, the cells were washed twice with PBS and then subjected to subsequent assays. The subsequent experiments adopted the same method to construct the oxidative stress model in vitro. The MitoTracker Red CMX Ros (C1035-250 μg, Beyotime Biotechnology, China) staining method was employed to assess the proportion of healthy mitochondria in the Mouse Leukaemic Monocyte Macrophage Cell Line (Raw264.7 cells). Cells were divided into the following groups: control, H_2_O_2_, CaAl-LDH + H_2_O_2_, and CALM + H_2_O_2_ (at 25, 50, and 100 μg mL−1). After treatment, the cells were washed with pre-warmed PBS and incubated with 100 nM MitoTracker Red CMXRos at 37°C for 30 min. After incubation, the cells were washed twice with PBS to remove the excess dye. Mitochondrial morphology was visualized using a fluorescence microscope (Olympus, Japan). The PK Mito Orange (PKMO) (PKMO-1, Nanjing Wobio Biotechnology Co., Ltd. China) staining method was used to examine mitochondrial morphology in the following groups: control, H_2_O_2_, CaAl-LDH + H_2_O_2_, and CALM + H_2_O_2_ (at 25, 50, and 100 μg mL−1). After treatment, cells were observed under a fluorescence microscope (Olympus, Japan). Fluoresce with an excitation wavelength of approximately 595 nm and an emission wavelength of approximately 620 nm. To measure mitochondrial membrane potential (MMP), the JC-1 dye (MT09, Dojindo, Japan) was employed. We seeded Raw264.7 cells into 96-well plates, dividing them into the following groups: control, H_2_O_2_, CaAl-LDH + H_2_O_2_, and CALM + H_2_O_2_ (at 25, 50, and 100 μg mL−1). Pre-prepared JC-1 working solution was added and incubate for 30–60 min at 37°C in a 5% CO_2_ incubator. After treatment, remove the supernatant and wash cells twice with HBSS. Finally, add Imaging Buffer Solution and observe cells under a fluorescence microscope (Olympus, Japan). JC-1 aggregates (representing polarized mitochondria) fluoresce red, with excitation at 488 nm and emission at 500-550 nm. JC-1 monomers (indicating depolarized mitochondria) fluoresce green, with excitation at 561 nm and emission at 560-610 nm. Red/green fluorescence ratio was analyzed using ImageJ. The ATP detection kit (S0026, Beyotime Biotechnology, China) was used to assess mitochondrial ATP production levels. After treatment, remove the culture medium and add lysis buffer to lyse the cells. Once the cells are fully lysed, centrifuge them at 12,000 g for 5 min at 4°C. Then, remove the supernatant and measure it using an enzyme-linked immunosorbent assay (Multiskan MK3, Thermo Fisher Scientific, USA) reader. To evaluate the effects of CALM on mitochondrial autophagy, the experimental treatments were conducted in the following four groups: control, H_2_O_2_, CaAl-LDH + H_2_O_2_, and CALM + H_2_O_2_ (at 25, 50, and 100 μg mL−1). Observe mitochondrial autophagy and lysosomal engagement using the Mitophagy Detection Kit (Dojindo, Japan).

### 4.10 Western blotting

Observation of mitochondrial fusion, fission and autophagy: Expression levels of OPA1, DRP1, Atg-7 and LC3 in raw264.7 were determined by Western blotting. Primary antibodies were applied to separated proteins. All polyvinylidene fluoride membranes were incubated with secondary antibody (goat anti-rabbit IgG horseradish peroxidase-labeled antibody). GAPDH served as the housekeeping gene.

### 4.11 In vitro anti-inflammatory capacity

Using the raw264.7 cells cultured as described above. The experiment comprised the following groups: control, H_2_O_2_, H_2_O_2_+IL-4, and H_2_O_2_+CALM. To evaluate the in vitro anti-inflammatory effects of CALM, we stimulated RAW264.7 macrophages with H_2_O_2_, inducing their polarization toward the pro-inflammatory M1 phenotype, which is characterized by increased intracellular iNOS expression. First, pretreat cells with CALM solution for 1 h. Then add H_2_O_2_ and incubate for 24 h. Discard the medium and collect the cells. Dilute the cell suspension with PBS to achieve a uniform concentration of 1×10^7^ cells/mL across all groups. Extract proteins from the cells using the repeated freeze-thaw method. INOS levels in cells were detected according to the iNOS ELISA kit instructions (S0021S, Beyotime Biotechnology, China). To further evaluate CALM's elimination effect on proinflammatory factors, we selected proinflammatory factors secreted under H_2_O_2_ stimulation (NO, TNF-α, and IL-6) for assessment. After culturing for 24 h according to the aforementioned grouping, collect the cell supernatants. Detect the concentrations of NO, TNF-α, and IL-6 in the supernatants following the steps outlined in the kit instructions (S0021S, Beyotime Biotechnology, China; MB-2868A, Jiangsu Meibiao Biotechnology Co., Ltd., China; MB-2899A, Jiangsu Meibiao Biotechnology Co., Ltd., China). Immunofluorescence staining allows for a more direct visualization of macrophage polarization. We seeded RAW264.7 cells in the logarithmic growth phase at a density of 3×10^5^ cells/well onto 24-well plates with coverslips. The experiment comprised the following groups: control, H_2_O_2_, H_2_O_2_+IL-4, and H_2_O_2_+CALM. After 24 h of incubation, staining was performed using a commercial kit, followed by observation under a fluorescence microscope.

### 4.12 In vitro osteogenesis capacity

Bone marrow mesenchymal stem cells (BMSCs) were isolated from osteoporotic Sprague-Dawley rats and incubated with low-glucose Dulbecco's Modified Eagle's Medium (DMEM) supplemented with 10% (v/v) fetal bovine serum (FBS, Gibco) at 37°C and 5% CO2 atmosphere. The experiment was divided into four treatment subgroups: four subgroups for treatment: control, H_2_O_2_, CaAl-LDH + H_2_O_2_, and CALM + H_2_O_2_. BMSCs were seeded in 96-well plates containing osteogenic induction medium (OIM) and treated with the respective materials for either 7 or 14 days. Alkaline phosphatase (ALP) staining was performed after 7 days to evaluate early osteogenic differentiation. The cells were washed three times with PBS, fixed with 4% paraformaldehyde for 15 min, and then incubated with ALP staining solution at room temperature for 30 min. After thorough washing with distilled water, observe the stained pores under the microscope. ALP enzymatic activity was quantified using a commercial ALP activity assay kit, following the manufacturer's instructions. After 14 days of induction, Alizarin Red S (ARS) staining was performed to assess late-stage mineralization. Cells were fixed with 4% paraformaldehyde for 15 min, rinsed with PBS, and stained with 0.2% ARS solution for 30 min at room temperature. Excess dye was removed by washing with distilled water, and stained nodules were imaged. For quantitative analysis, 10% cetylpyridinium chloride was added to solubilize calcium-bound dye, and absorbance was measured at 570 nm using a microplate reader. To measure intracellular calcium levels in BMSCs, the aforementioned cultured cells were treated with the Fluo4-AM assay kit (Dojindo, Japan). After treatment, wash cells three times with HBSS solution to thoroughly remove residual Fluo4-AM working solution. Then add HBSS solution to cover the cells. Incubate at 37°C for approximately 20-30 min to ensure complete deesterification of AM bodies within cells. Examine cells using confocal or fluorescence microscopy with excitation at 494 nm and emission at 516 nm.

To evaluate the osteoimmunomodulatory capacity of the CALM coating, macrophage-conditioned medium (CM) was prepared. Briefly, RAW264.7 cells were subjected to different treatments (H_2_O_2_, CaAl-LDH + H_2_O_2_, and CALM + H_2_O_2_) for 24 h. Subsequently, the culture supernatants were collected and centrifuged at 2000 rpm for 10 min to remove cellular debris. The macrophage-derived CM was then mixed with fresh OIM at a volumetric ratio of 1:1. BMSCs were seeded in 24-well plates and cultured with the respective mixture media. To evaluate early osteogenic differentiation, ALP and ARS staining were performed according to the protocols described above.

### 4.13 qRT-PCR and western blotting

For macrophage-reprogramming-related genes and hypoxia-related genes, including Nos2, Tnf, Arg1, Cd206, Hif1a, and Glut1, total RNA was extracted from RAW264.7 macrophages after the indicated treatments. For osteogenesis-related genes, including Osterix, P2X7, Runx2, Bmp2, Col1a1, and Ocn, total RNA was extracted from BMSCs after osteogenic induction. The extracted RNA was reverse-transcribed into complementary DNA (cDNA). Quantitative real-time PCR (qRT-PCR) was performed using a SYBR Green qRT-PCR kit (TaKaRa, Dalian, China) and an ABI StepOnePlus Real-Time PCR System. Gapdh was used as the internal reference gene for normalization. For hypoxia-related qRT-PCR assays, RAW264.7 macrophages were cultured under 1% O_2_ and treated with H_2_O_2_ in the presence or absence of CALM (100 μg mL−1) for 24 h. The groups included normoxic control, hypoxia + H_2_O_2_, and hypoxia + H_2_O_2_ + CALM.

Total cellular proteins were extracted using a protein extraction kit. The proteins were mixed with loading buffer and denatured by boiling. Denatured samples were loaded onto SDS-polyacrylamide gels for electrophoretic separation. Following separation, proteins were transferred onto a polyvinylidene fluoride membrane (0.45 μm pore size; Millipore, Bedford, Massachusetts, USA) and blocked for 0.5 h with 5% skimmed milk powder diluted in Tris-buffered saline containing Tween-20. Then, they were incubated at 4°C overnight with anti-BMP antibody (1:1000; ab14933, Abcam), anti-BGP antibody (1:1000; InvivoGen, France), anti-P-PI3K antibody (1:1000; ab191606, Abcam), anti PI3K antibody (1:1000; ab302958, Abcam), anti-P-Akt antibody (1:1000; ab38449, Abcam), anti-Akt antibody (1:1000; ab179463, Abcam), anti-P-GSK3*β* antibody (1:1000; ab32391, Abcam), anti-GSK3*β* antibody (1:1000; ab179463, Abcam), and anti-GAPDH anti body (1:1000; ab313650, Abcam). After washing, membranes were incubated with horseradish peroxidase (HRP)-conjugated secondary antibodies (1:5000, Santa Cruz Biotechnology) for 1 h at room temperature. Protein bands were visualized using an enhanced chemiluminescence detection system (Thermo Fisher Scientific, Pierce, Rockford, IL, USA). Relative gene expression was calculated using the 2^-ΔΔCq^ method, with GAPDH serving as the endogenous control.

### 4.14 Preparation of rat femoral fracture model

All animal procedures were approved by the Committee on Ethics of Biomedicine, Second Military Medical University (82402776). Ninety-six Sprague-Dawley rats (SD rats, 6 months old, all female) were obtained from Second Military Medical University (82402776) with an average weight of 240 g. Before implantation, the bilateral ovaries were removed and the rats were fed for another 3 months. Micro-CT was used to confirm that osteoporotic SD rats were constructed successfully. Prior to the femoral fracture surgery, all rats were strictly baseline-matched for body weight to eliminate allocation bias. The rats were randomly allocated to four groups (n = 24 per group): bare titanium Kirschner wire (Ti), HA@Ti, CaAl-LDH@HA@Ti and CALM@HA@Ti. Different Ti-based implants (diameter: 1.0 mm; length: 30 mm) were inserted into the distal end of the femur of osteoporotic SD rats. Briefly, osteoporotic SD rats were anesthetized via injecting 2.5% 2,2,2-Tribromoethanol into abdominal cavity. Under general anesthesia and aseptic conditions, a 1 cm lateral incision was made on the right knee joint. The subcutaneous tissue and muscle were incised sequentially, and the patella was displaced medially to expose the femoral intercondylar fossa and distal femur. Reamer (1.5 mm in diameter) was inserted into the medullary cavity via the distal femur and advanced to the proximal femur. After removal of the reamer, the medullary cavity was flushed with saline. Subsequently, Kirschner wires (1.0 mm in diameter) were implanted, and the incision was closed in layers. A specialized fracture modeling device (RuiTaiMos Biotech, China) was used to induce a femoral fracture. The fracture model and internal implants were confirmed using X-ray imaging. Finally, 0.1 mL penicillin (5000U/mL) was injected into muscle on the left leg in the first three days after surgery.

### 4.15 In vivo osteogenic properties and biosafety

The harvested femur specimens were scanned using a high-resolution micro-CT system (Skyscan 1172, Bruker microCT, Kontich, Belgium). To ensure data accuracy, the system underwent daily calibration using a standard hydroxyapatite (HA) phantom. Scanning was performed with the following optimized parameters: a source voltage of 70 kV, a current of 114 μA, an isotropic spatial resolution of 18.2 μm, and an integration time of 250 ms per projection. Three-dimensional (3D) reconstruction of the fracture site was subsequently conducted using dedicated analysis software (CT-Analyzer, V1.17.7.2, Bruker microCT). The region of interest (ROI) was established to quantify bone regeneration at the fracture site. The VOI featured a total longitudinal height of 10 mm, centered on the fracture midpoint and extending 5 mm both proximally and distally along the femoral axis. In the coordinate system, the analysis span was precisely mapped from an origin of 4.95 mm to an endpoint of 14.97 mm along the vertical Z-axis. This comprehensive VOI was designed to encapsulate all external, internal, and intramedullary callus formations. To ensure the accuracy of the bone morphometric data, metal-induced beam-hardening artifacts arising from the titanium implants were rigorously suppressed using manual threshold-based segmentation. Within the defined VOI, the following primary parameters were quantified: bone mineral density (BMD, mg/cm^3^), bone volume fraction (BV/TV, %), and trabecular number (Tb.N, 1/mm). Sequential fluorescent labeling was employed to evaluate the rate of new bone formation. Rats euthanized at week 4 post-surgery received intraperitoneal injections of calcein (5 mg mL−1, 5 mL kg−1 body weight) at week 2 post-surgery and alizarin red S (2.5 mg mL−1, 2.5 mg kg−1 body weight) at week 3 post-surgery. Rats sacrificed at 8 weeks post-surgery received calcein and alizarin red S injections at 4 and 6 weeks post-surgery, respectively. Penicillin (0.1 mL, 5000 U/mL) was administered three days after each drug injection. Collected femur specimens were fixed in 10% paraformaldehyde for one week, dehydrated with ethanol, and sectioned parallel to the femoral long axis using a hard tissue microtome (Exakt, Germany). Sections were initially cut at 150 μm thickness and subsequently ground to 40 μm. Fluorescence imaging was performed using a laser scanning confocal microscope (LSCM) (Nikon, Japan) with calcein excitation/emission wavelengths of 488/500–550 nm and alizarin red S at 543/580–670 nm. Mineralization deposition rate (MAR) was quantified using Image Pro 6.0 software.

Hard tissue sections stained with haematoxylin and eosin (HE) and Masson's trichrome stain (Masson) were examined for new bone formation using a panoramic slide scanner. Biomechanical stability was assessed via a three-point bending test, with femoral specimens secured in the biomechanical testing machine's three-point bending fixture. Compressive loading was applied at 2 mm/min towards the fracture site to determine the maximum load the femur could withstand. To evaluate the in vivo biocompatibility of CALM@HA@Ti, three rats were randomly selected from each group at weeks 2 and 4 post-surgery. After anesthesia, blood samples were collected from the heart for routine blood tests and assessment of liver and kidney function. These parameters were compared with those of normal, non-operated rats to ensure the biocompatibility of the material. To explicitly evaluate the in vivo immunomodulatory effects of the coatings at the fracture site, immunofluorescence staining was performed on the decalcified callus tissue sections. Following deparaffinization, rehydration, and antigen retrieval, the tissue sections were blocked and incubated overnight at 4°C with primary antibodies against iNOS and CD206. Subsequently, the sections were incubated with corresponding fluorophore-conjugated secondary antibodies in the dark, and the cell nuclei were counterstained with DAPI. The immunofluorescence images were captured using a confocal laser scanning microscope (or fluorescence microscope), and the fluorescence intensity/positive cell areas were quantitatively analyzed using ImageJ software to assess the localized M1/M2 macrophage polarization state.

Additionally, to assess the mechanical stability and adherence of the composite coatings during surgical handling and implantation, the weight variations of the Kirschner wires were rigorously monitored. A high-precision electronic balance (accuracy: 0.1 mg) was employed to measure the weights of the implants prior to the operation (W_pre_) and on the second post-operative day (W_po_) following retrieval.

$$\text{Attrition}\;\text{rate}\;\left(\%\right)=\frac{W_{\text{pre}}-W_{\text{po}}}{W_{\text{pre}}-W_{\text{blank}}}\times 100\%$$

where W_pre_, W_po_ and W_blank_ indicate the weights of Kirschner wires with different coatings before the operation, at the second day after surgery, and pure Kirschner wires without any coating, respectively.

### 4.16 RNA extraction and library construction

Total RNA was isolated and purified using TRIzol reagent (Invitrogen, Carlsbad, CA, USA) following the manufacturer's procedure. The RNA amount and purity of each sample was quantified using NanoDrop ND-1000 (NanoDrop, Wilmington, DE, USA). The RNA integrity was assessed by Bioanalyzer 2100 (Agilent, CA, USA) with RIN number >7.0, and confirmed by electrophoresis with denaturing agarose gel. Poly (A) RNA is purified from 1 μg total RNA using Dynabeads Oligo (dT)25-61005 (Thermo Fisher, CA, USA) using two rounds of purification. Then the poly(A) RNA was fragmented into small pieces using Magnesium RNA Fragmentation Module (NEB, cat. e6150, USA) under 94°C 5-7min. Then the cleaved RNA fragments were reverse-transcribed to create the cDNA by SuperScript™ II Reverse Transcriptase (Invitrogen, cat. 1896649, USA), which were next used to synthesise U-labeled second-stranded DNAs with *E. coli* DNA polymerase I (NEB, cat. m0209, USA), RNase H (NEB, cat. m0297, USA) and dUTP Solution (Thermo Fisher, cat. R0133, USA). An A-base is then added to the blunt ends of each strand, preparing them for ligation to the indexed adapters. Each adapter contains a T-base overhang for ligating the adapter to the A-tailed fragmented DNA. Single- or dual-index adapters are ligated to the fragments, and size selection was performed with AMPureXP beads. After the heat-labile UDG enzyme (NEB, cat. m0280, USA) treatment of the U-labeled second-stranded DNAs, the ligated products are amplified with PCR by the following conditions: initial denaturation at 95°C for 3 min; 8 cycles of denaturation at 98°C for 15 s, annealing at 60°C for 15 s, and extension at 72°C for 30 s; and then final extension at 72°C for 5 min. The average insert size for the final cDNA library was 300±50 bp. At last, we performed the 2×150bp paired-end sequencing (PE150) on an illumina Novaseq™ 6000 (LC-Bio Technology CO., Ltd., Hangzhou, China) following the vendor's recommended protocol.

### 4.17 Bioinformatics analysis of RNA-seq

Fastp software (https://github.com/OpenGene/fastp) was used to remove the reads that contained adaptor contamination, low quality bases and undetermined bases with default parameter. Then sequence quality was also verified using fastp. We used HISAT2 (ftp.ensembl.org/pub/release-112/fasta/mus_musculus/dna/) to map reads to the reference genome of *Mus musculus* GRCm39. The mapped reads of each sample were assembled using StringTie (https://ccb.jhu.edu/software/stringtie) with default parameters. Then, all transcriptomes from all samples were merged to reconstruct a comprehensive transcriptome using gffcompare (https://github.com/gpertea/gffcompare/). After the final transcriptome was generated, StringTie was used to estimate the expression levels of all transcripts. StringTie was used to estimate mRNA expression levels by calculating FPKM (FPKM = [total_exon_fragments/mapped_reads(millions) × exon_length(kB)]). The differentially expressed mRNAs were selected with fold change >2 or fold change <0.5 and with parametric F-test comparing nested linear models (p value < 0.05) by R package edgeR (https://bioconductor.org/packages/release/bioc/html/edgeR.html).

### 4.18 Statistical analysis

All experiments are performed with at least three independent replicates. Data are presented as the mean ± standard deviation (SD). Statistical significance between the experimental group and the control group is calculated with a two-tailed Student's t-test and One-way analysis of variance (ANOVA). ∗ denotes a statistical significance (∗p < 0.05, ∗∗p < 0.01, ∗∗∗p < 0.001, and ∗∗∗∗p ＜ 0.0001) between the data of the experimental group and the control group. All statistical analyses were performed by GraphPad Prism 10.1.0.178 (GraphPad Software, Boston, MA, USA).

## CRediT authorship contribution statement

**Bo Yuan:** Conceptualization, Data curation, Funding acquisition, Investigation, Methodology, Writing – original draft, Writing – review & editing. **Jia Fu:** Data curation, Formal analysis, Investigation, Methodology, Software, Writing – original draft, Writing – review & editing. **Yin Zhao:** Data curation, Formal analysis, Funding acquisition, Investigation, Software, Writing – original draft, Writing – review & editing. **Gang Zheng:** Data curation, Formal analysis, Investigation, Software, Writing – original draft. **Han Lin:** Conceptualization, Project administration, Resources, Software, Supervision, Writing – review & editing. **Xiongsheng Chen:** Conceptualization, Project administration, Software, Supervision, Writing – review & editing. **Xiang Guo:** Conceptualization, Funding acquisition, Project administration, Resources, Supervision, Writing – review & editing. **Jianlin Shi:** Project administration, Supervision, Writing – review & editing.

## Declaration of competing interest

The authors declare that they have no known competing financial interests or personal relationships that could have appeared to influence the work reported in this paper.

## Data Availability

Data will be made available on request.
