# Supplementary material for: Tri-modal nanocatalytic microenvironment regulations for macrophage reprogramming and osteoporotic fracture healing promotion
Source: Mater Today Bio. 2026 Jun 23;39:103389. doi: 10.1016/j.mtbio.2026.103389 (PMC13324845; doi:10.1016/j.mtbio.2026.103389)
Supplement: Multimedia component 1 [file mmc1.docx]

**Tri-modal Nanocatalytic Microenvironment Regulations for Macrophage Reprogramming and Osteoporotic Fracture Healing Promotion**


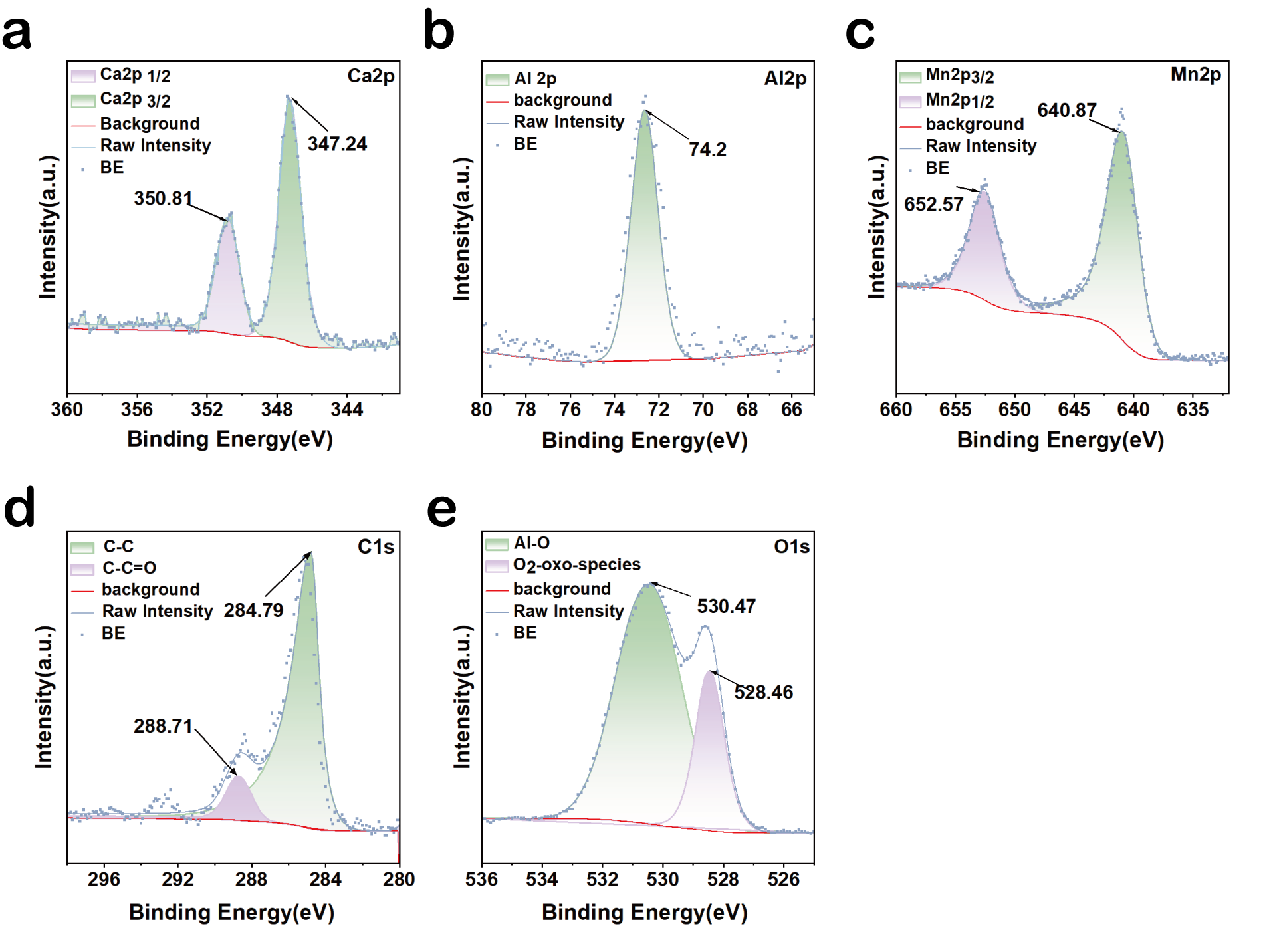


**Figure S1**. XPS spectra of CALM. a) Ca 2p, b) Al 2p, c) Mn 2p, d) C 1s, and e) O 1s.


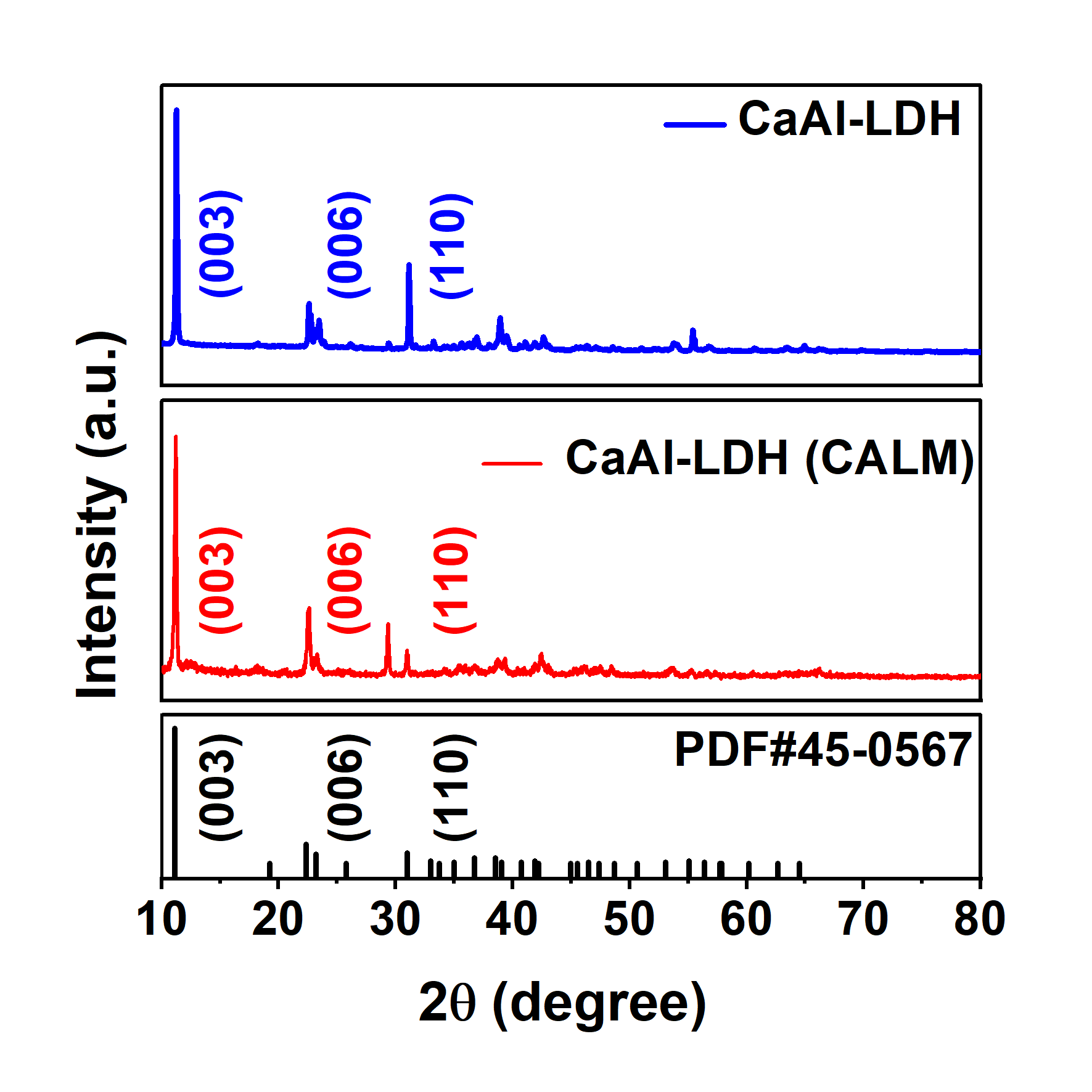


**Figure S2.** XRD patterns of CaAl-LDH (blue) and CALM (red).


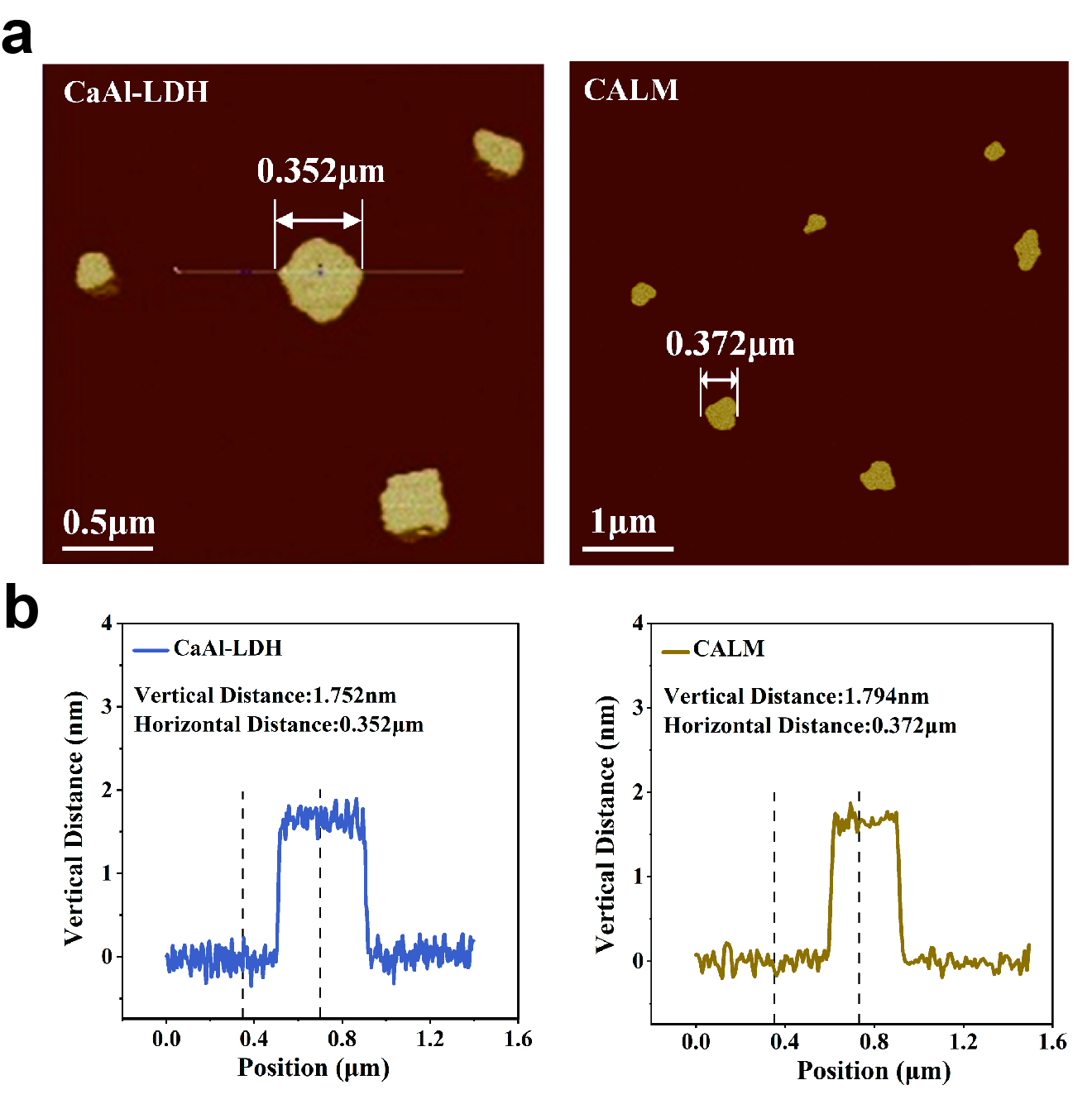


**Figure S3.** a) 2D AFM image of CaAl-LDH and CALM. b) Measurement of vertical and horizontal distance of CaAl-LDH and CALM.


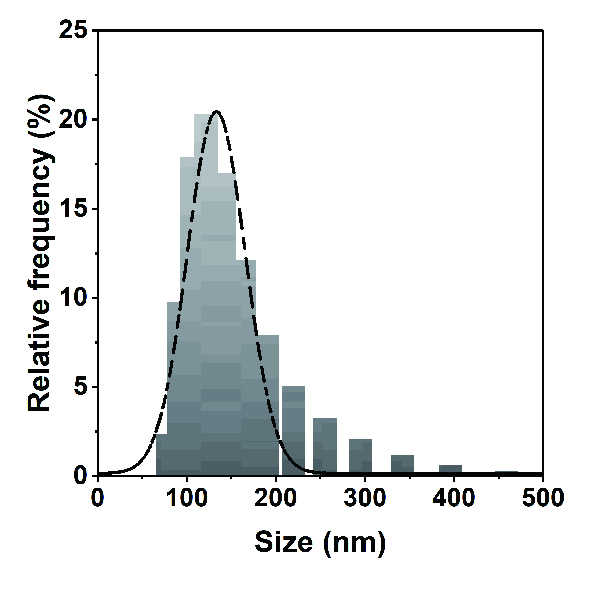


**Figure S4**. Measurement of particle size of CALM.


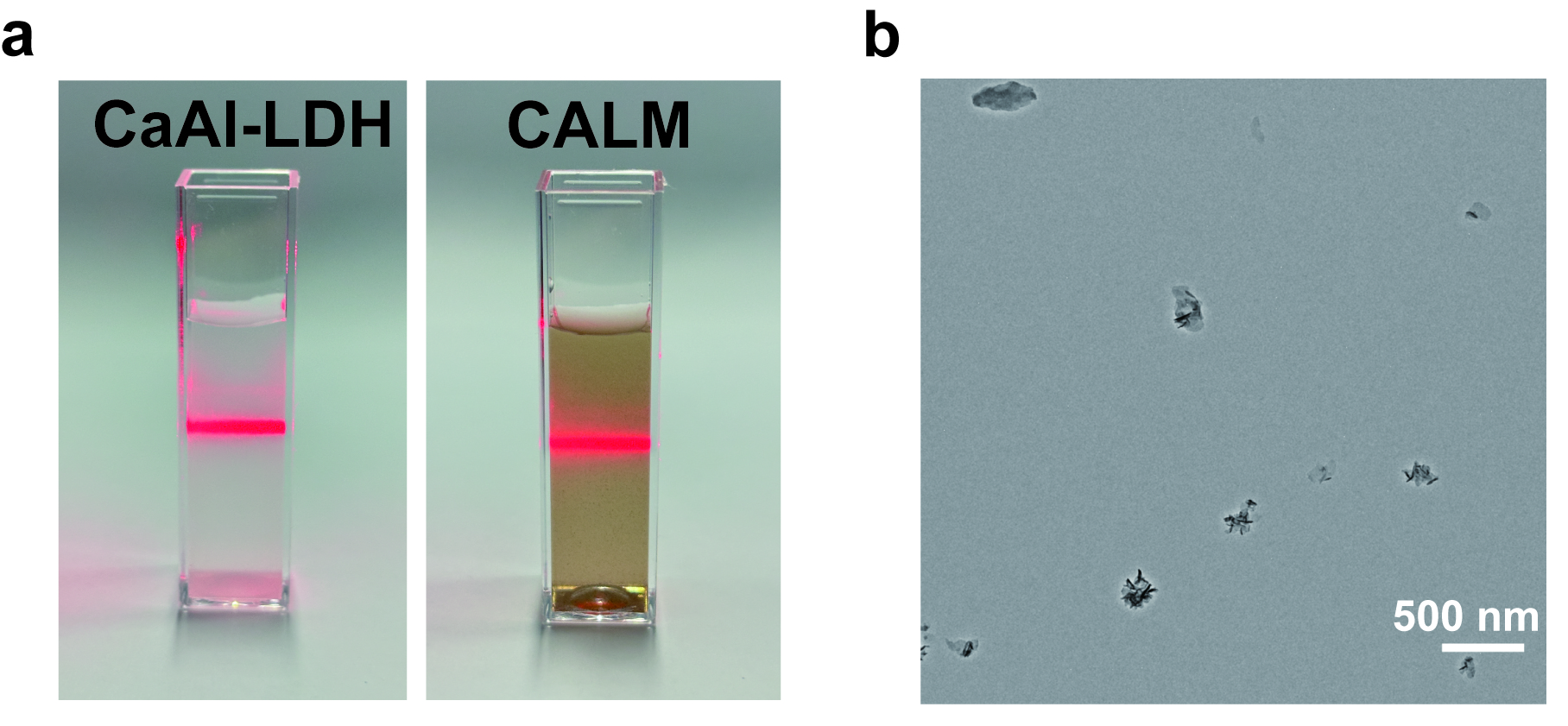


**Figure S5.** a) Photographs of the Tyndall effect showing a visible laser track through the suspension. b) Transmission electron microscopy of CALM.


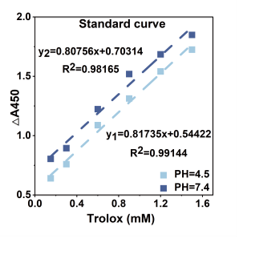


**Figure S6.** Standard curve of ROS-scavenging activities of CALM nanosheets toward multiple free radicals


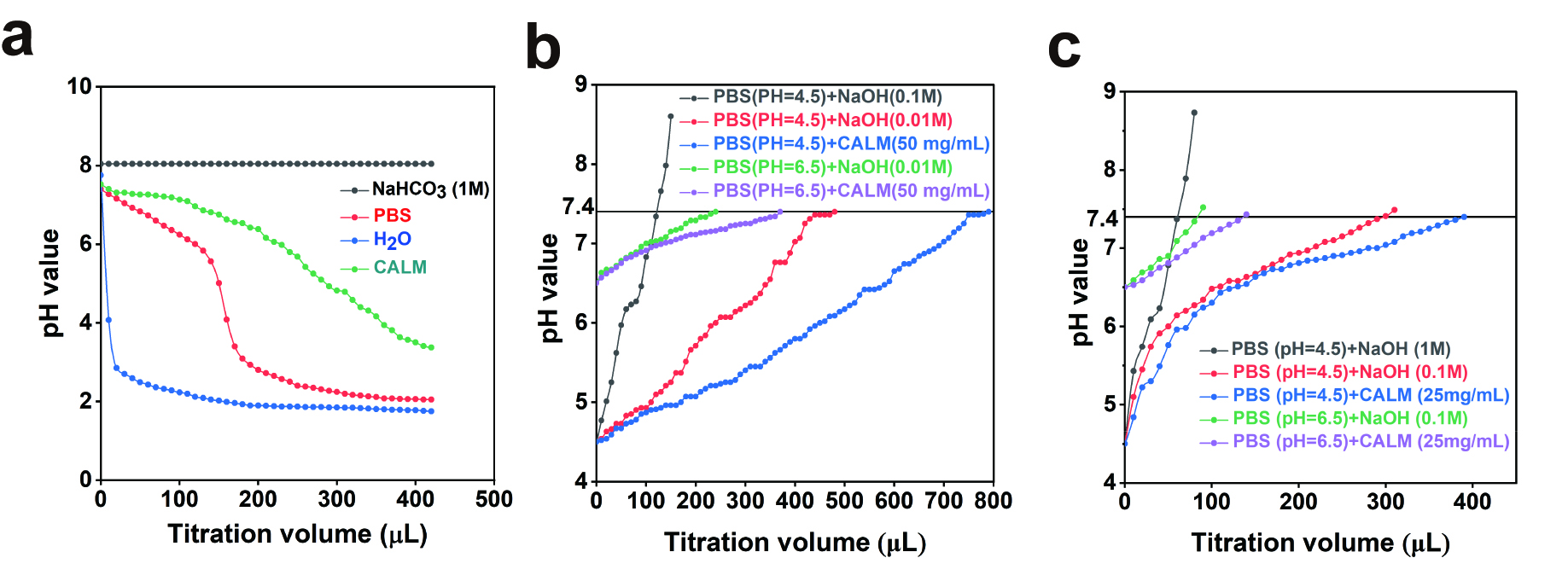


**Figure S7**. a) pH monitoring during the titration of 1% HCl into different solutions (NaHCO_3_, PBS, H_2_O, and CALM). b) pH monitoring during the titration of 0.1 M NaOH, 0.01 M NaOH, and 50 mg/mL CALM into acidic PBS (pH 4.5 or 6.5). c) pH monitoring during the titration of 1 M NaOH, 0.1 M NaOH, and 25 mg/mL CALM into acidic PBS (pH 4.5 or 6.5).


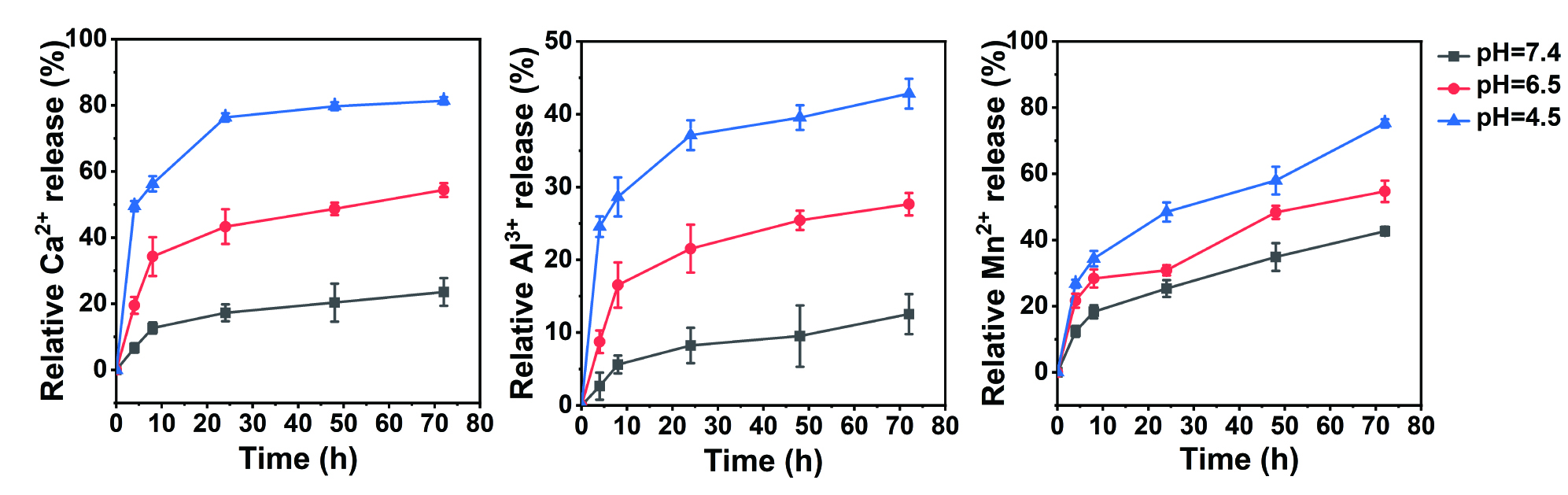


**Figure S8.** Cumulative release percentages of Ca^2+^, Al^3+^, and Mn^2+^ from CALM under pH-responsive degradation conditions.


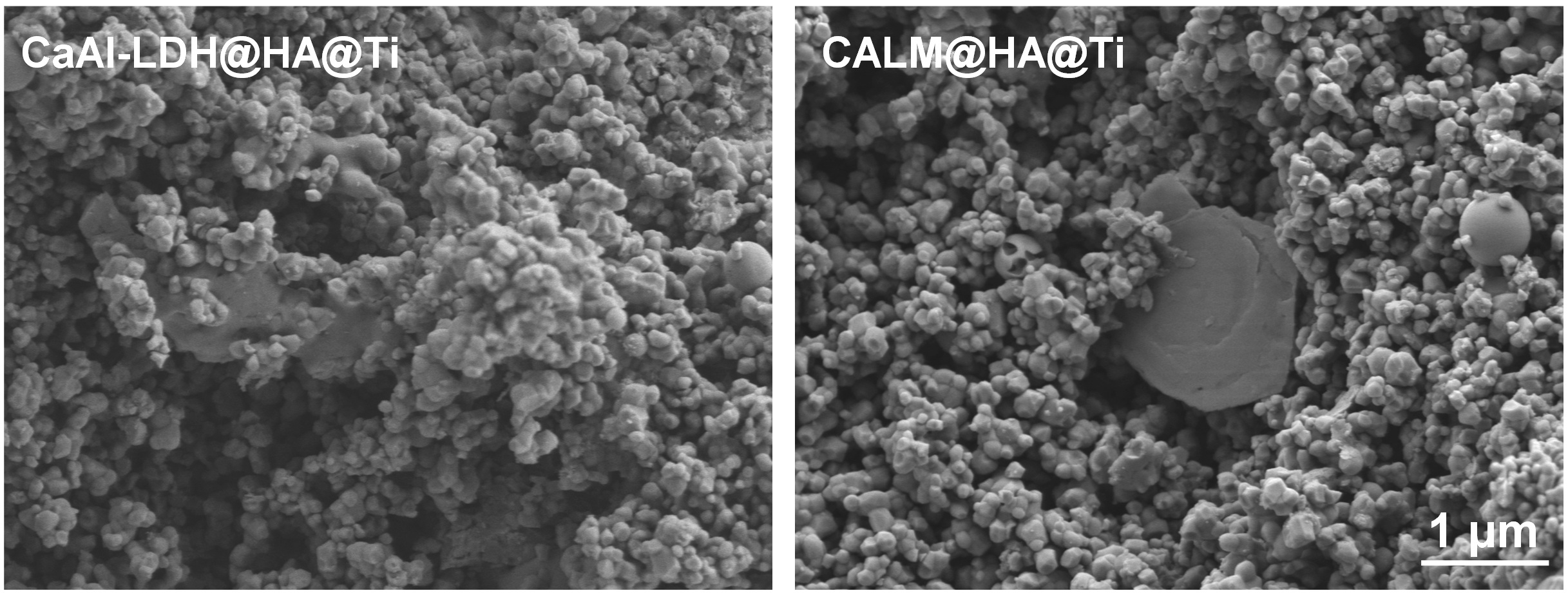


**Figure S9.** SEM of CaAl-LDH@HA@Ti and CALM@HA@Ti after immersion in PBS for 14 days.


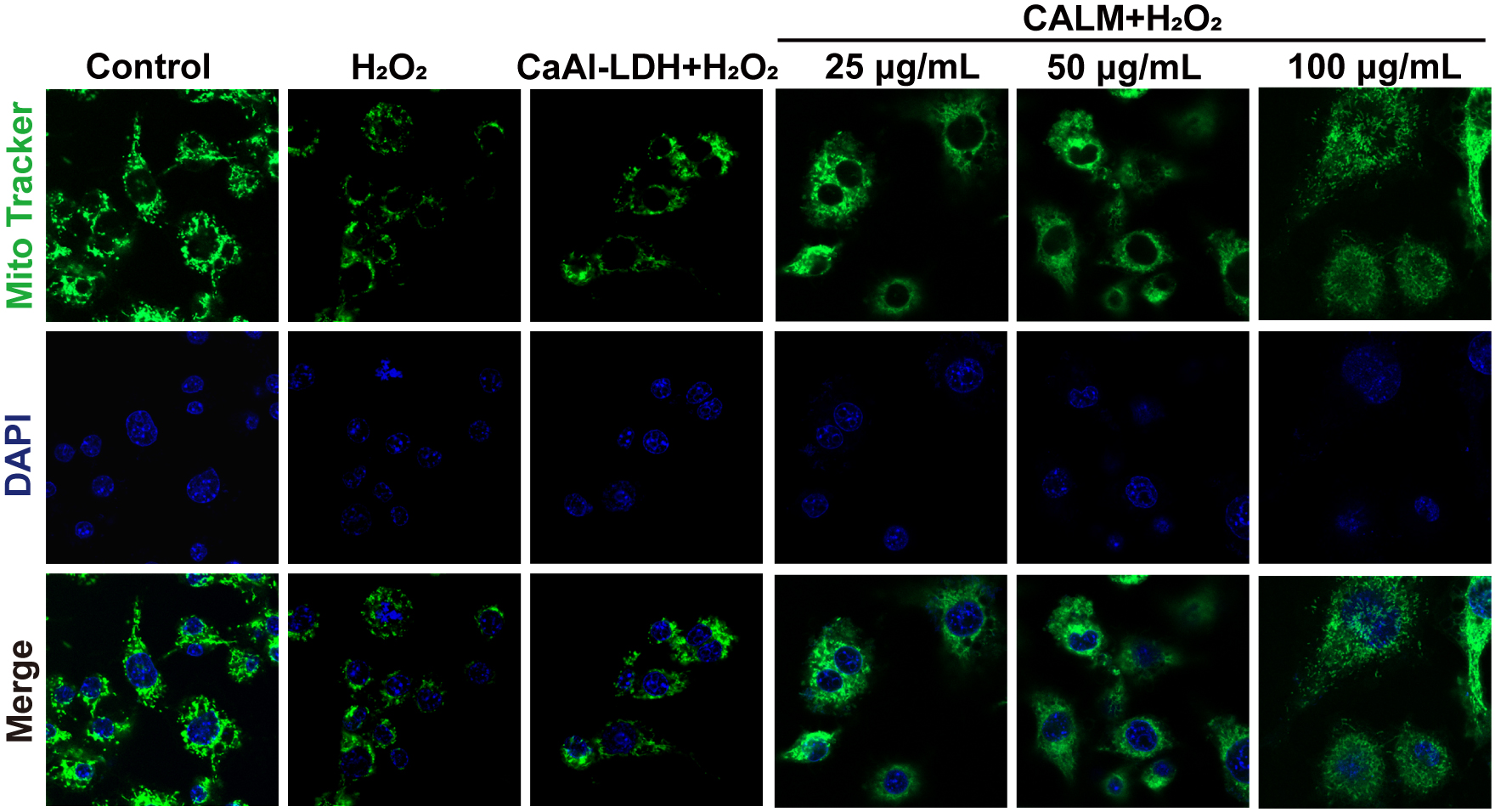


**Figure S10.** Representative images of mitochondrial architecture labeled by Mito Tracker (green fluorescence).


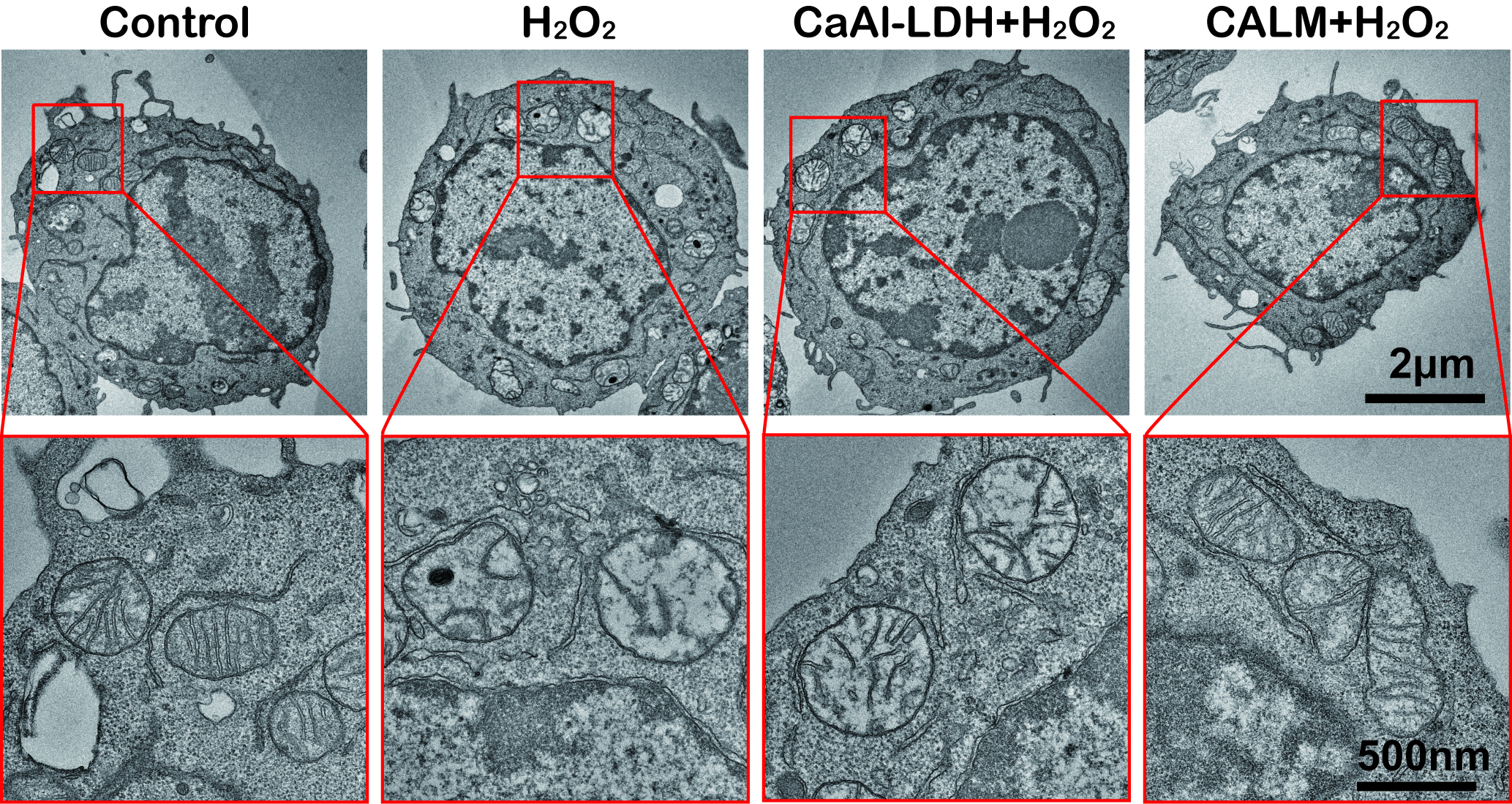


**Figure S11**. Mitochondrial morphology map captured by transmission electron microscopy.


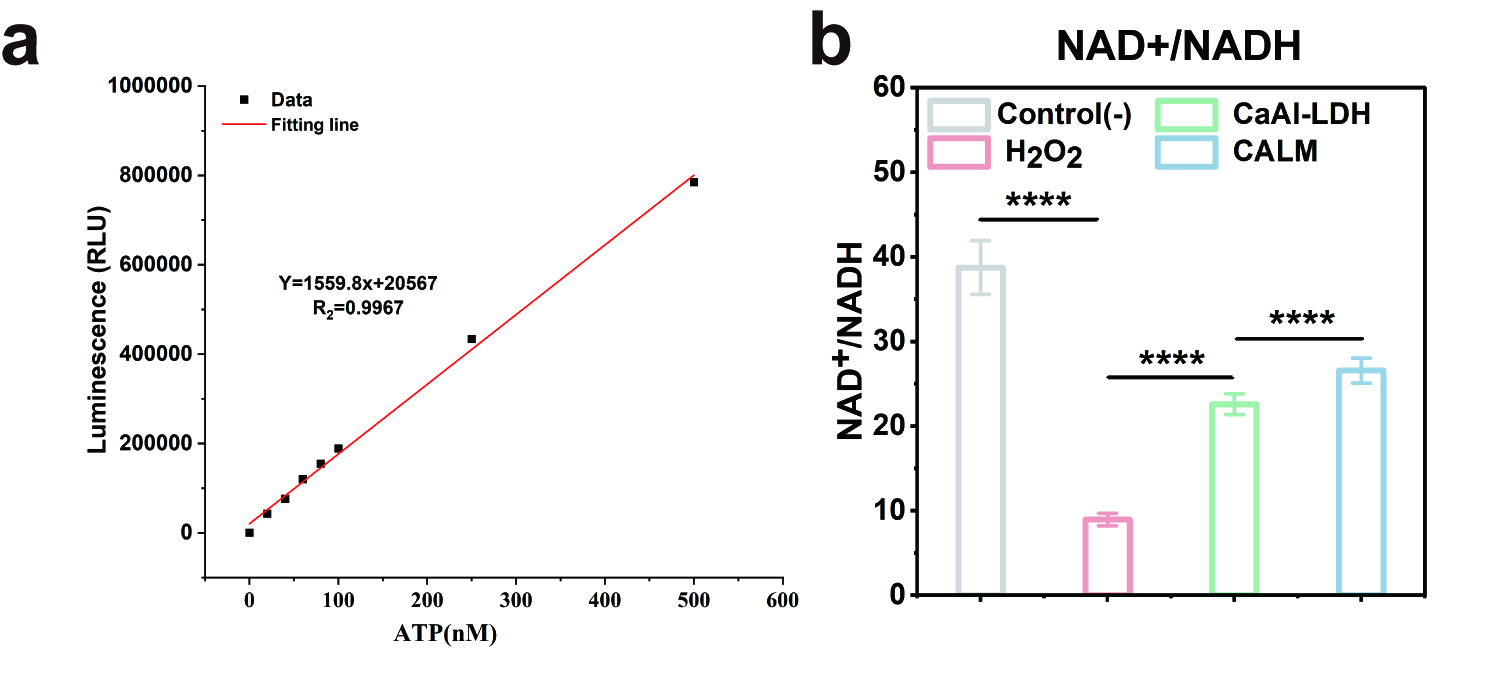


**Figure S12.** a) ATP standard curve and b) NAD+/NADH ratio.





**Figure S13.** Relative mRNA expression levels of Hif1a and Glut1 in RAW264.7 macrophages under normoxic control, hypoxia + H_2_O_2_, and hypoxia + H_2_O_2_ + CALM conditions.


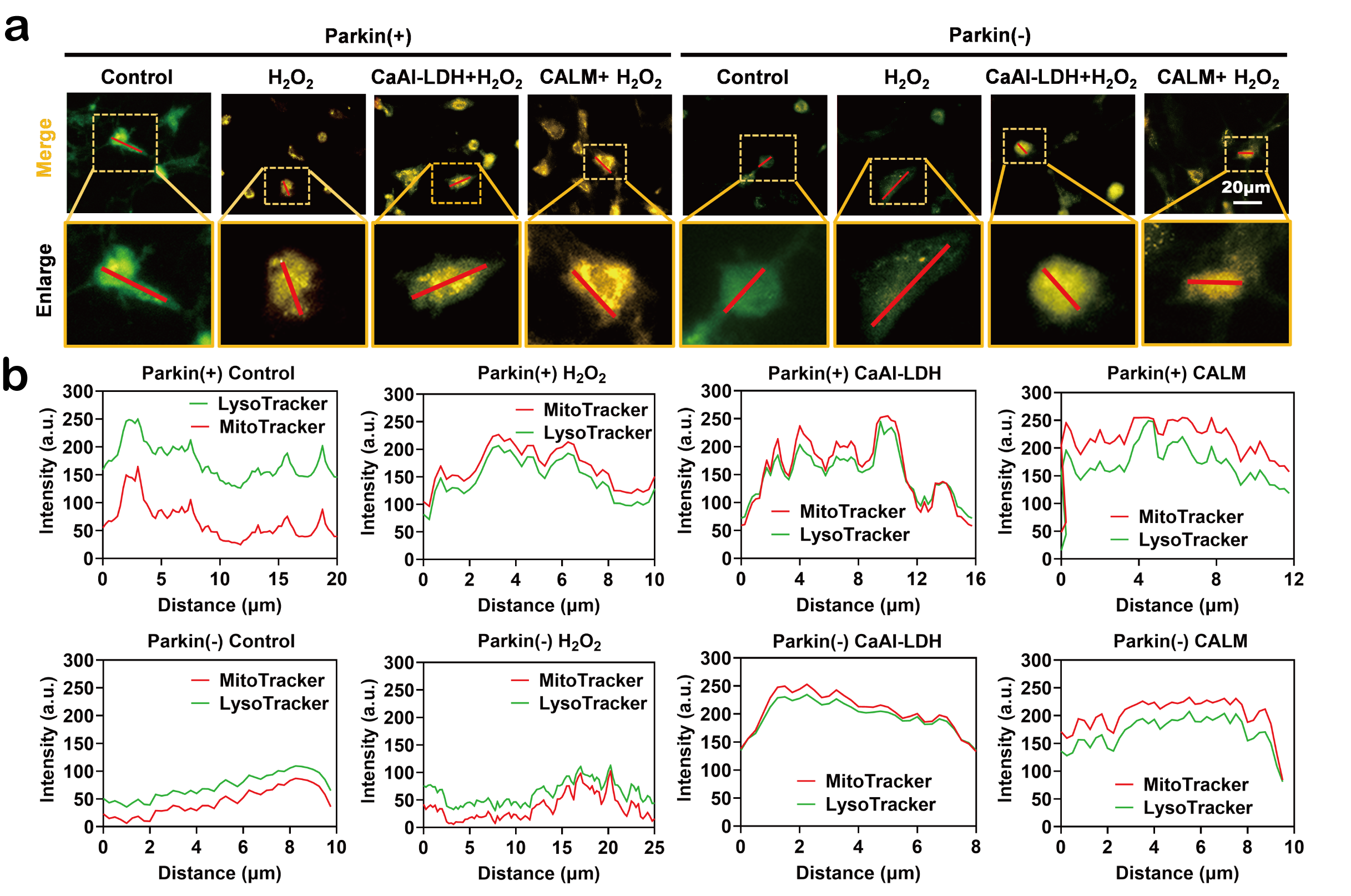


**Figure S14.** Confocal laser microscopy images of macrophages and line-scanning profiles of green and red fluorescence from the selected regions.


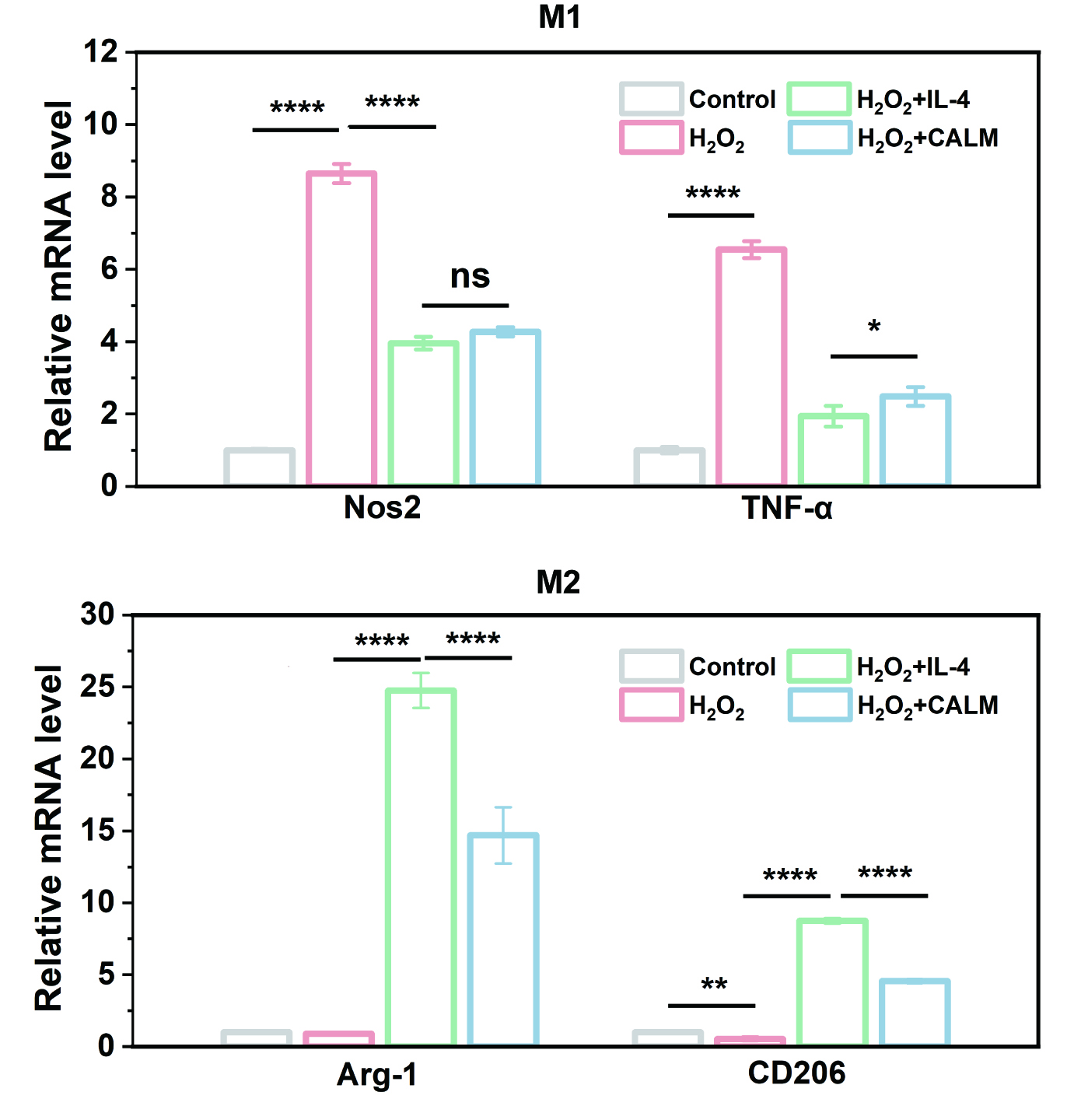


**Figure S15**. Quantitative analysis of macrophage polarization-related gene expression in RAW264.7 cells under different treatments.


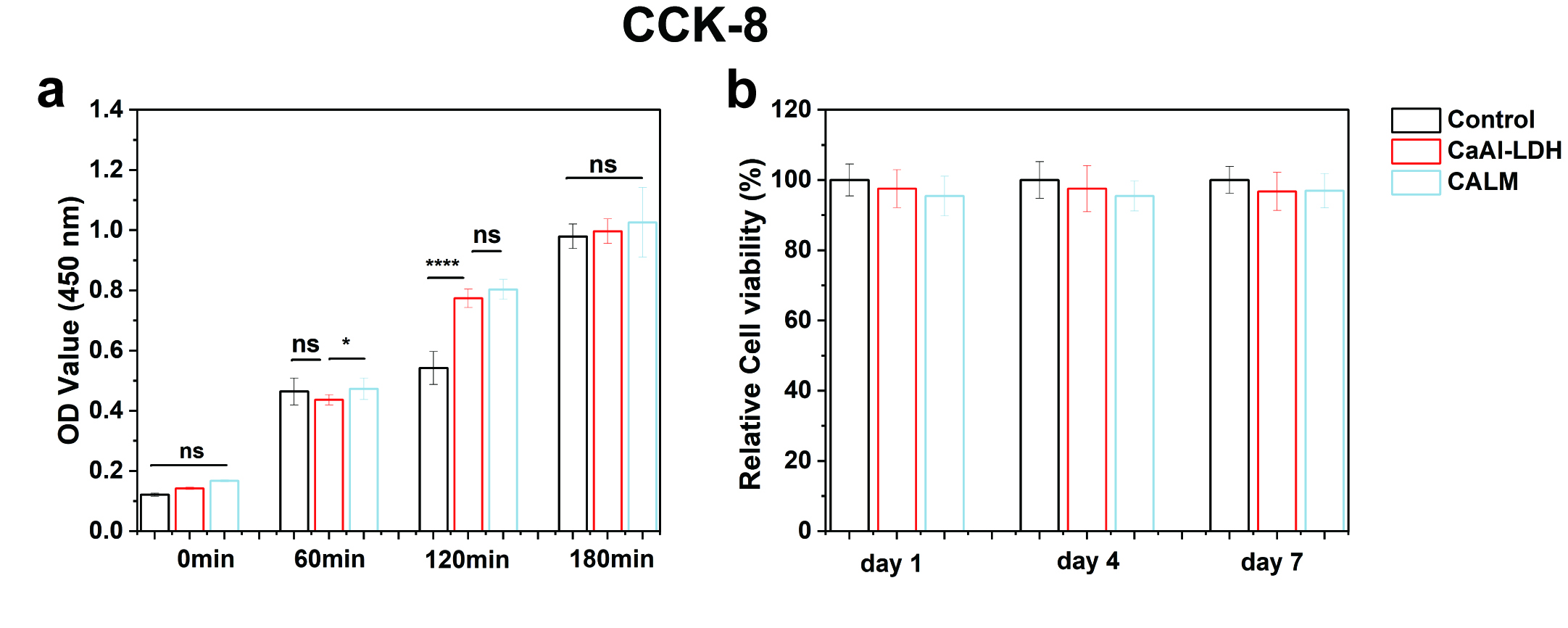


**Figure S16.** a) OD values from the CCK-8 assay after 3 h of culture in different groups. b) Relative cell viability over 7 days.


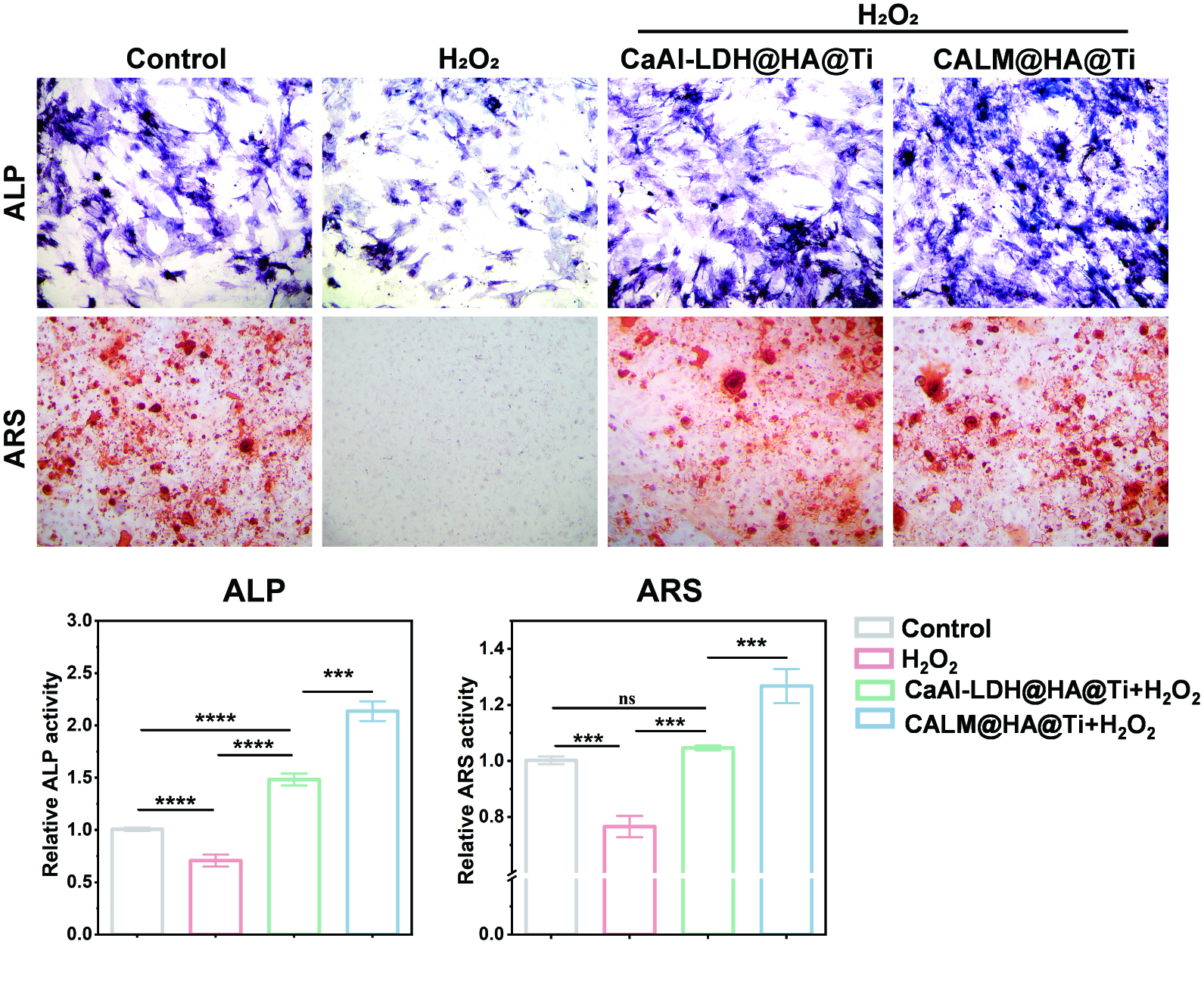


**Figure S17.** Representative images of ALP staining, ARS staining, and corresponding quantitative analysis.


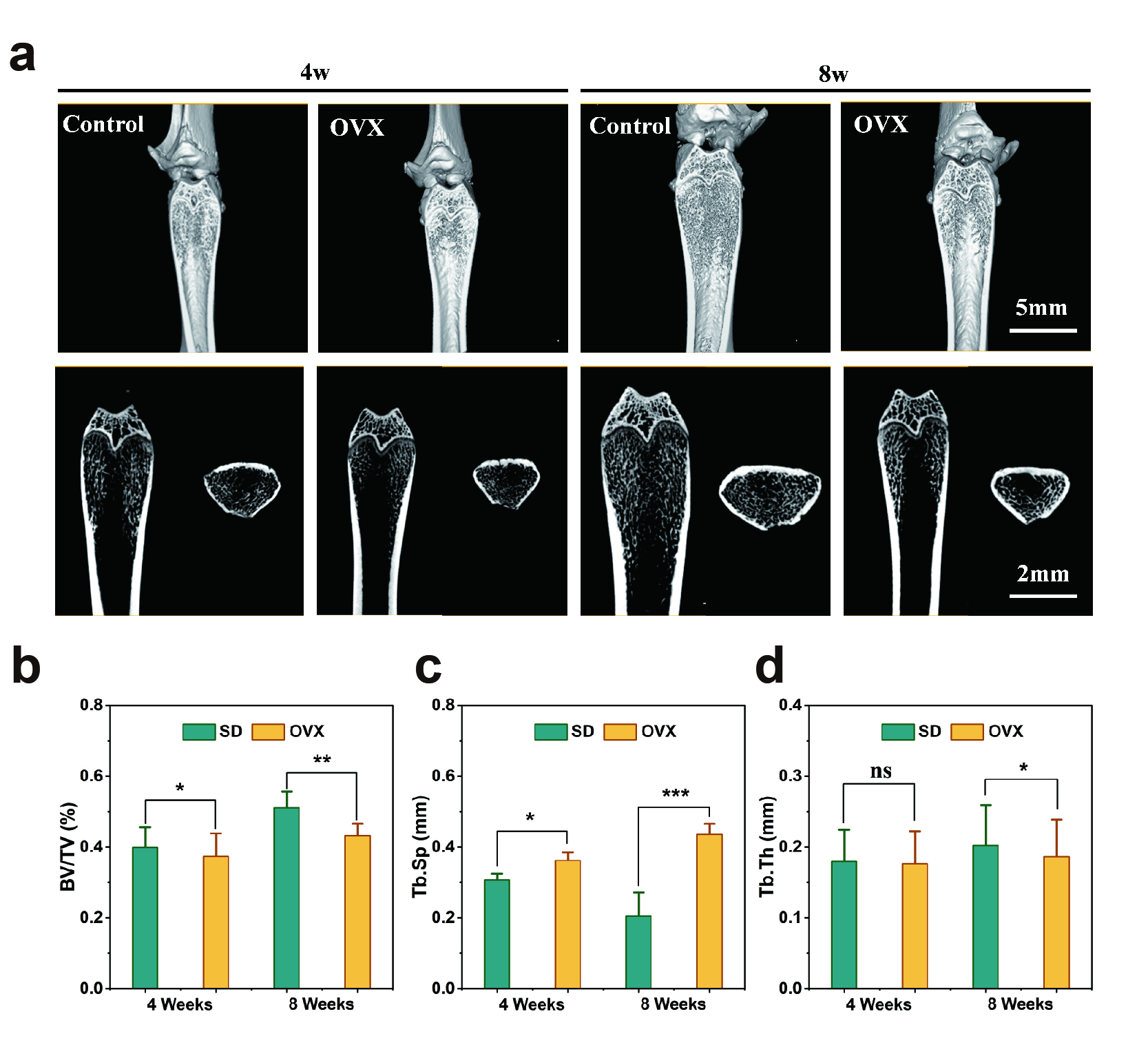


**Figure S18.** Establishment and validation of an osteoporotic rat model. a) Representative micro-CT images of osteoporotic bones at 4 and 8 weeks post-surgery across different groups. c-d) Quantitative analysis of BV/TV, Tb. Sp, and Tb. Th of cancellous bone based on micro-CT data.


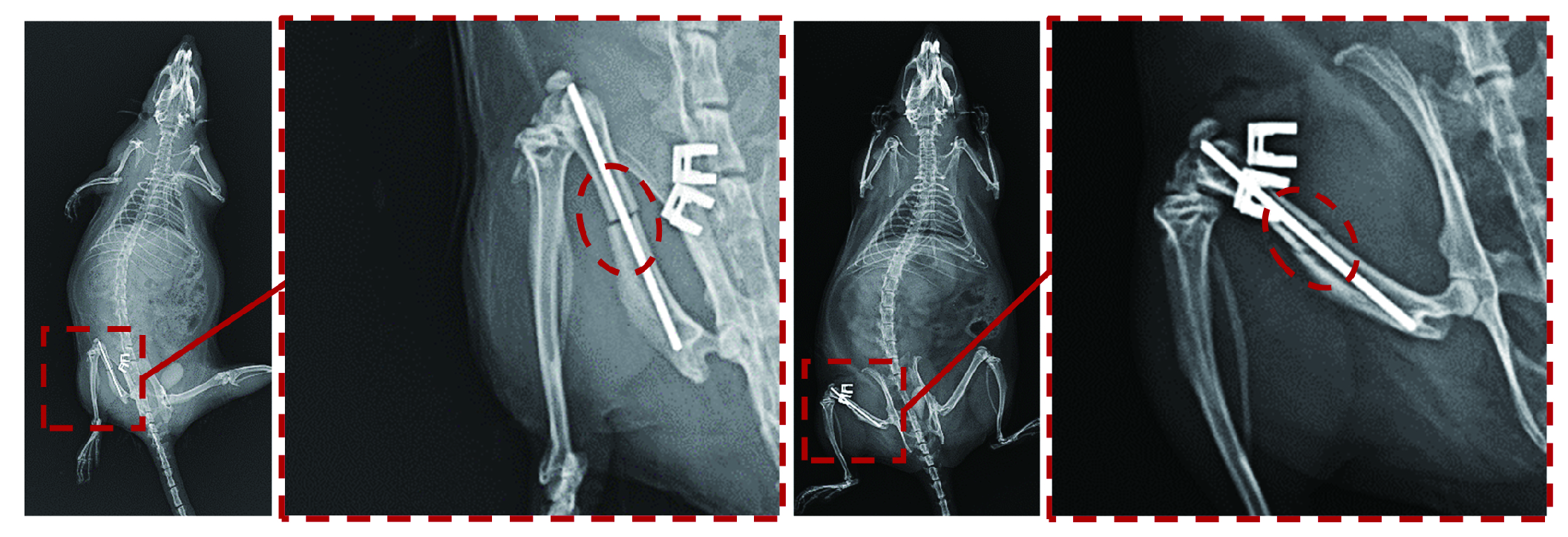


**Figure S19.** Establishment and validation of a linear femur fracture based on X-ray.


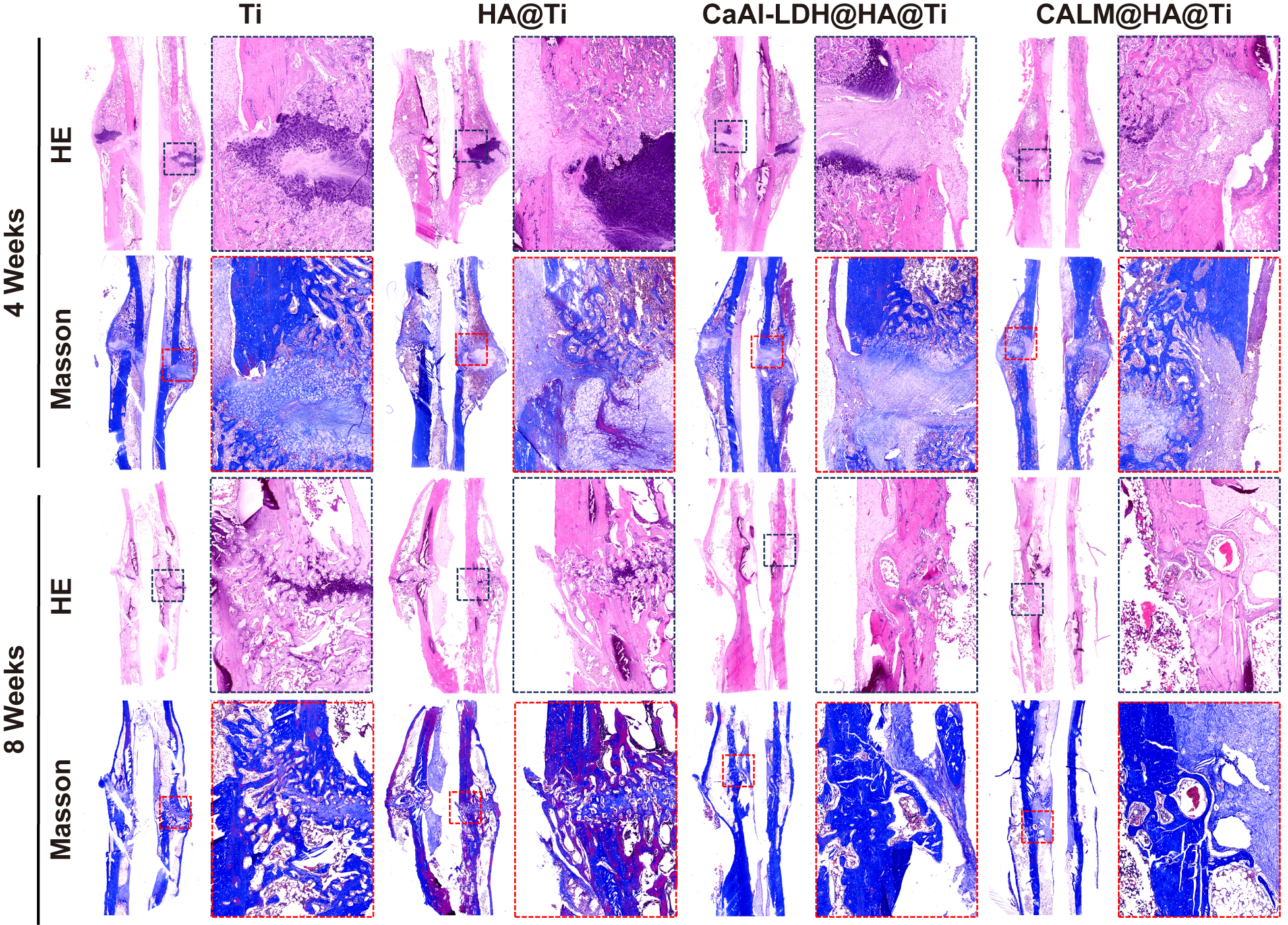


**Figure S20.** Histological analysis of HE and Masson staining in different groups.


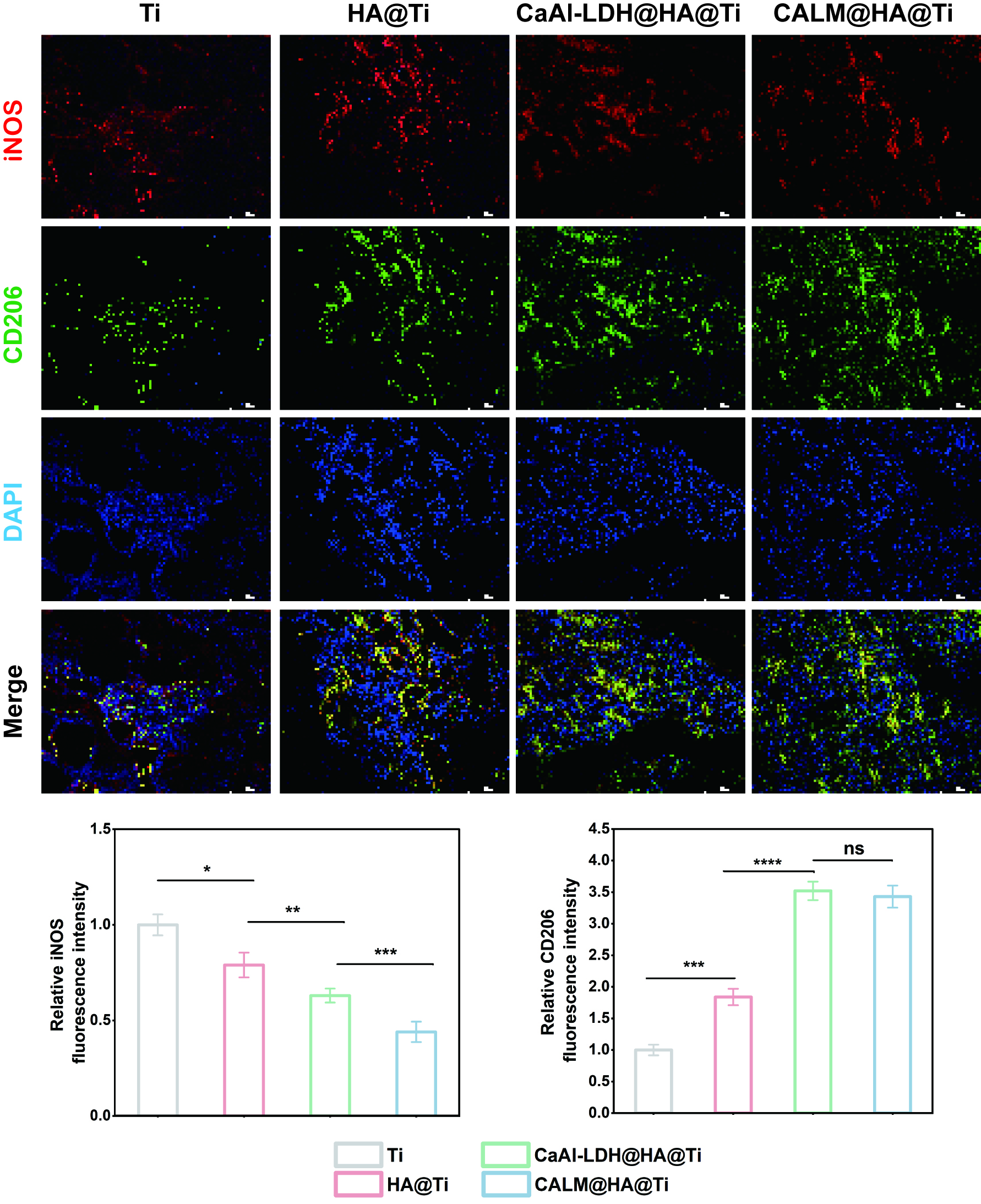


**Figure S21.** Immunofluorescence staining of the M2 macrophage marker CD206 (green) and the M1 macrophage marker iNOS (red), with corresponding fluorescence intensity quantified from the immunofluorescence staining.


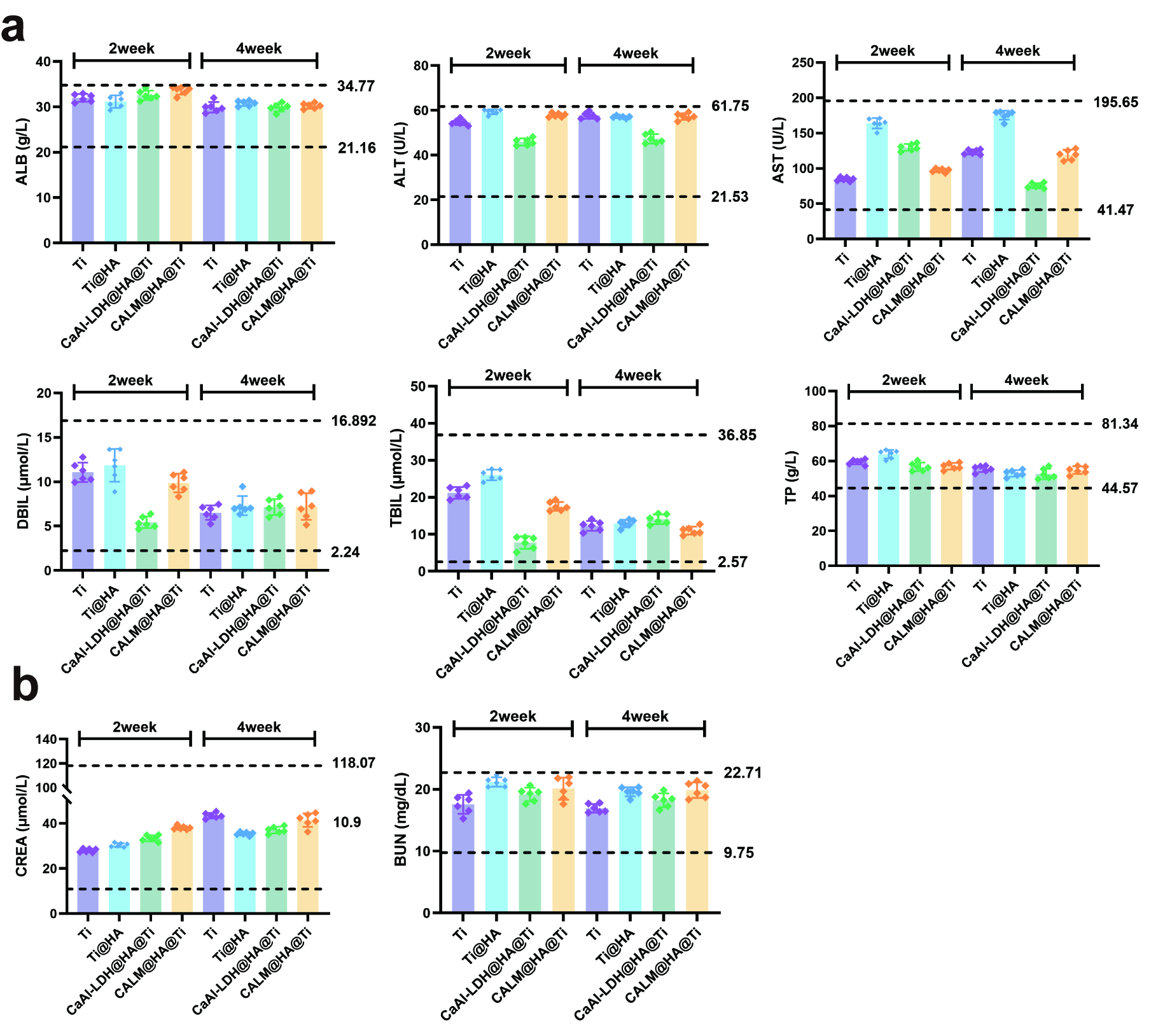


**Figure S22.** a) Liver and b) kidney function of SD rats at different time points after the surgery.





**Figure S23.** Attrition rates in different groups during surgical handling and implantation.


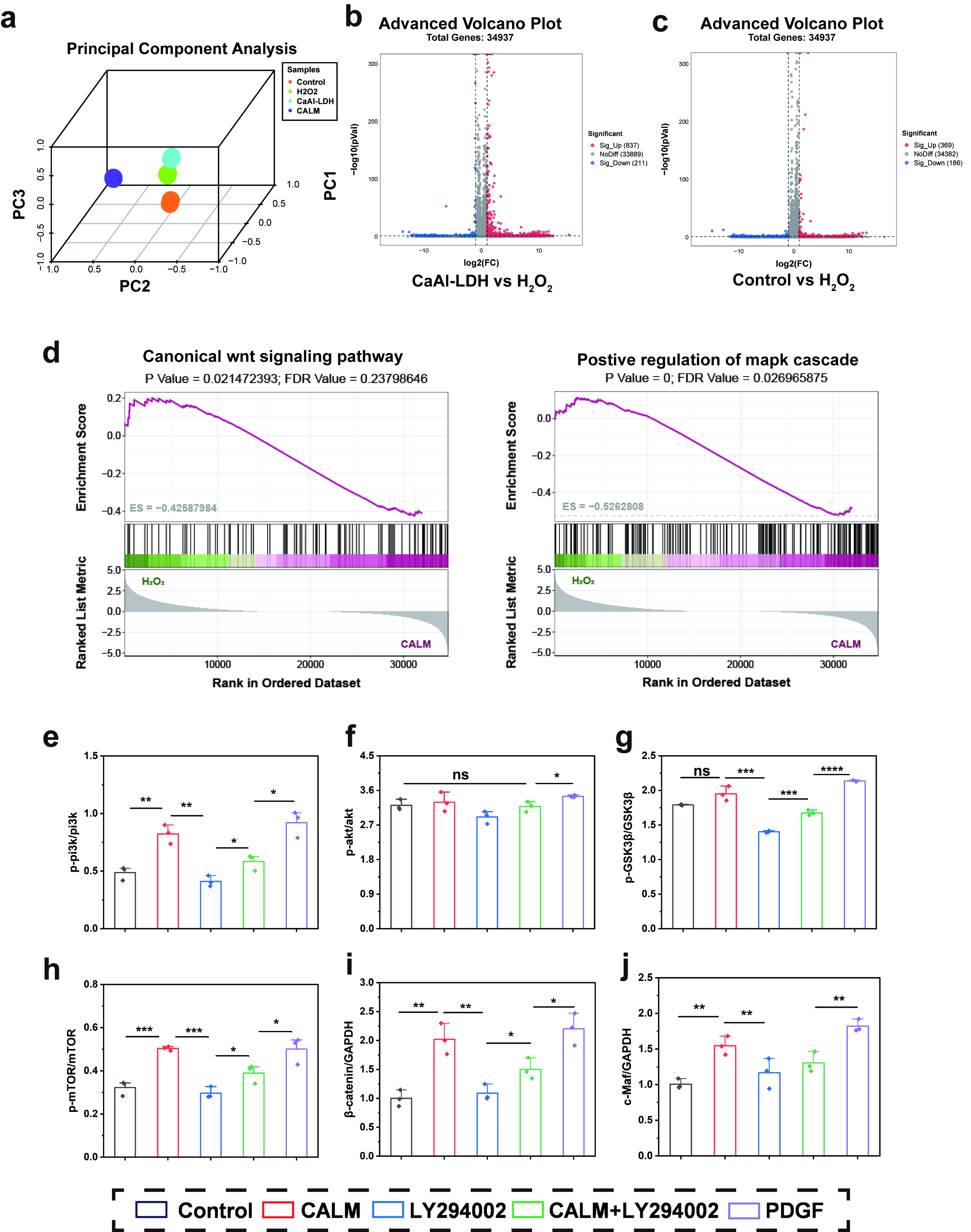


**Figure S24.** a) Principal component analysis (PCA). b, c) Volcano plots of differentially expressed genes for the CaAl-LDH vs H2O2 and Control vs H2O2 comparisons. d) GSEA enrichment analysis. e-j) Quantitative analysis of Western blot results.
